# Causes of death and infant mortality rates among full-term births in the United States between 2010 and 2012: An observational study

**DOI:** 10.1371/journal.pmed.1002531

**Published:** 2018-03-20

**Authors:** Neha Bairoliya, Günther Fink

**Affiliations:** 1 Harvard Center for Population and Development Studies, Cambridge, Massachusetts, United States of America; 2 Swiss Tropical and Public Health Institute, Basel, Switzerland; 3 University of Basel, Basel, Switzerland; Cambridge University, UNITED KINGDOM

## Abstract

**Background:**

While the high prevalence of preterm births and its impact on infant mortality in the US have been widely acknowledged, recent data suggest that even full-term births in the US face substantially higher mortality risks compared to European countries with low infant mortality rates. In this paper, we use the most recent birth records in the US to more closely analyze the primary causes underlying mortality rates among full-term births.

**Methods and findings:**

Linked birth and death records for the period 2010–2012 were used to identify the state- and cause-specific burden of infant mortality among full-term infants (born at 37–42 weeks of gestation). Multivariable logistic models were used to assess the extent to which state-level differences in full-term infant mortality (FTIM) were attributable to observed differences in maternal and birth characteristics. Random effects models were used to assess the relative contribution of state-level variation to FTIM. Hypothetical mortality outcomes were computed under the assumption that all states could achieve the survival rates of the best-performing states. A total of 10,175,481 infants born full-term in the US between January 1, 2010, and December 31, 2012, were analyzed. FTIM rate (FTIMR) was 2.2 per 1,000 live births overall, and ranged between 1.29 (Connecticut, 95% CI 1.08, 1.53) and 3.77 (Mississippi, 95% CI 3.39, 4.19) at the state level. Zero states reached the rates reported in the 6 low-mortality European countries analyzed (FTIMR < 1.25), and 13 states had FTIMR > 2.75. Sudden unexpected death in infancy (SUDI) accounted for 43% of FTIM; congenital malformations and perinatal conditions accounted for 31% and 11.3% of FTIM, respectively. The largest mortality differentials between states with good and states with poor FTIMR were found for SUDI, with particularly large risk differentials for deaths due to sudden infant death syndrome (SIDS) (odds ratio [OR] 2.52, 95% CI 1.86, 3.42) and suffocation (OR 4.40, 95% CI 3.71, 5.21). Even though these mortality differences were partially explained by state-level differences in maternal education, race, and maternal health, substantial state-level variation in infant mortality remained in fully adjusted models (SIDS OR 1.45, suffocation OR 2.92). The extent to which these state differentials are due to differential antenatal care standards as well as differential access to health services could not be determined due to data limitations. Overall, our estimates suggest that infant mortality could be reduced by 4,003 deaths (95% CI 2,284, 5,587) annually if all states were to achieve the mortality levels of the best-performing state in each cause-of-death category. Key limitations of the analysis are that information on termination rates at the state level was not available, and that causes of deaths may have been coded differentially across states.

**Conclusions:**

More than 7,000 full-term infants die in the US each year. The results presented in this paper suggest that a substantial share of these deaths may be preventable. Potential improvements seem particularly large for SUDI, where very low rates have been achieved in a few states while average mortality rates remain high in most other areas. Given the high mortality burden due to SIDS and suffocation, policy efforts to promote compliance with recommended sleeping arrangements could be an effective first step in this direction.

## Abstract 2

Using state-level birth and death records, Neha Bairoliya identify state- and cause specific burden of infant mortality among full-term infants in the United States, and uncover opportunities for improvement.

## Author summary

**Why was this study done?:**

- High infant mortality rates in the US compared to other high-income countries have been well documented in the literature.
- Most of this literature primarily attributes high infant mortality in the US to the high rates of prematurity.
- Relatively little is known regarding the survival of infants born full-term.

**What did the researchers do and find?:**

- We compared state-level mortality rates among full-term infants in the US to that of 6 European countries with low mortality rates.
- We showed that infants born full-term in the US face 50%–200% higher risks of infant mortality compared to these European countries.
- We found that the largest proportion of infant deaths among children born full-term in the US was due to sudden unexpected deaths of infants, which comprised both sudden infant death syndrome and other unexpected causes such as suffocation and violence.

**What do these findings mean?:**

- Major improvements in full-term infant mortality (through increases in full-term infant survival and increases in pregnancy terminations) seem possible in the US.
- More research is needed to identify the most effective policies to achieve this objective.

## Introduction

Despite some progress made in recent years, infant mortality rates in the US continue to be high compared to other high-income countries [1]. According to the latest estimates, the US currently ranks 44th among 199 countries of all income levels, with an infant mortality rate of 5.6 deaths per 1,000 live births in 2015, about 3 times the rate observed for countries at the very top of the ranking [1].

**Fig 1:**
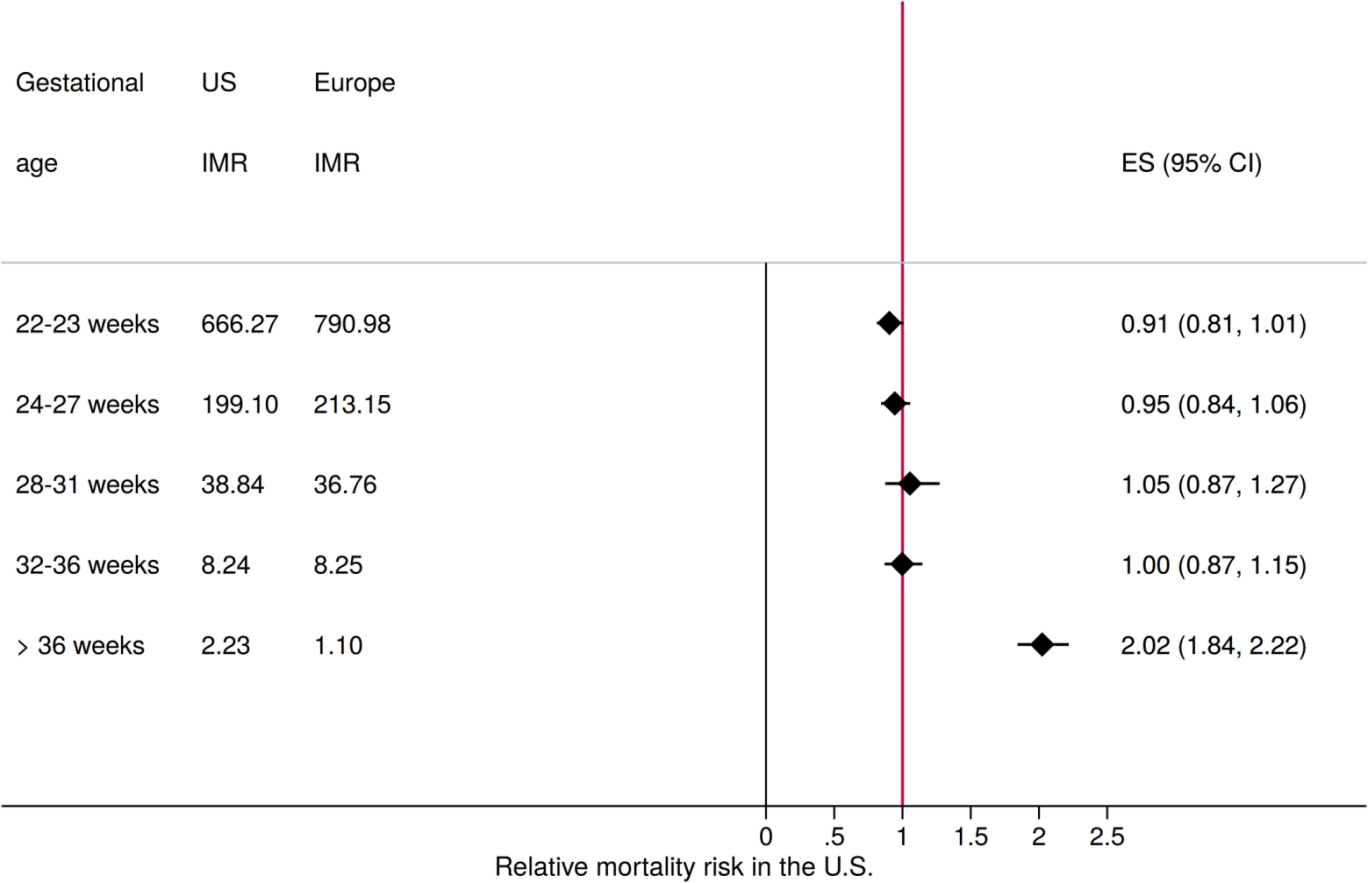
Relative mortality risk in the US and Europe by gestational age category. The figure shows infant mortality risk (IMR) in the US compared to the average rate observed in Austria, Denmark, Finland, Norway, Sweden, and Switzerland for the year 2010. Sources: Euro-Peristat, US birth and death records, author calculations. Gestational age in both the Euro-Peristat and US data is based on the best obstetrical estimate available, which in most cases corresponds to first trimester ultrasound. ES, effect size.

While the high rates of prematurity and prematurity-related mortality in the US have been well documented in the literature [2,3], the US performs comparably to other high-income countries when it comes to the survival of preterm infants. Fig 1 compares gestation-specific mortality rates in the US and 6 leading European countries (in terms of low infant mortality rates) with data available for 2010. On average, infant mortality appeared to be very similar for premature births in the US and in these European countries. The same was not true for children born after 36 weeks of gestation, where children born in the US faced more than twice the mortality risk of children in European countries with low infant mortality rates (odds ratio [OR] 2.02, 95% CI 1.84, 2.22). A recent US Centers for Disease Control and Prevention (CDC) report suggests that this mortality gap among full-term births now accounts for almost 50% of the infant mortality gap between Sweden and the US [4].

In this study, we used complete and geocoded birth records from the period 2010–2012 to better understand the high burden of mortality among full-term infants in the US. We identified the main causes underlying the high mortality rates among full-term infants overall in the aggregate data in a first step, and then explored differences in actual and potential birth outcomes across US states in a second step. By first reviewing the causes of death in this population, we could identify the main risk factors for infants in this generally low-risk population, and could clearly distinguish the relative importance of preexisting conditions such as malformations relative to perinatal and post-neonatal conditions (those arising in the 28–364 days after birth). In order to provide a better sense of feasible outcomes in this population, we estimated and compared cause-specific full-term mortality rates at the state level both unconditional and conditional on maternal characteristics. While these state-level comparisons did not allow us to identify the specific reasons why certain states have particularly high rates of mortality, they did allow us to identify areas where major improvements were possible in principle.

## Methods

### Study design

The study was designed as a cross-sectional study using birth and death records of all infants born in the US between January 1, 2010, and December 31, 2012. No pre-analysis plan was developed for this study. The main objective of the project was to identify the primary causes underlying the high infant mortality rates observed in the US nationally as well as at the state level.

### Data sources

Linked birth and death records including restricted geographic identifiers were obtained from the National Center for Health Statistics (NCHS) for the years 2010 to 2012. All infants in the birth and death records could be directly linked to the geographic identifiers in these datasets (100% match rate). Additional data for Fig 1 were downloaded from the Euro-Peristat webpage at http://www.europeristat.com/our-indicators/euro-peristat-perinatal-health-indicators-2010.html.

### Outcome measures

Our primary outcome measure of interest was the infant mortality rate among full-term births defined as the number of deaths per 1,000 children born alive between 37 and 42 weeks of gestation within the first year of their life. For the purpose of this study, we used the traditional definition of full-term, which includes early-term (37 and 38 weeks), full-term (39 and 40 weeks), late-term (41 weeks), and some post-term (42 weeks) births according to the more recent definition of the American College of Obstetricians and Gynecologists Committee on Obstetric Practice [5]. To adjust for differential outcomes in this relatively wide 5-week gestational window, we controlled for differences in gestational age by including binary indicators for gestational age category (37 weeks, 38 weeks, 41 weeks, 42 weeks) in our multivariable analysis, using the more narrow, revised full-term definition (39 and 40 weeks) as our reference group. Gestational age was computed by the NCHS based on last menstrual period reported by the mother. To ensure gestational age was not measured differentially across states, we compared prematurity rates with rates of low birth weight in the full sample at the state level. The correlation of these measures at the state level was 0.97; the strong alignment between birth weight and reported gestational age is further supported by the descriptive statistics provided in S1 Table.

Causes of death for all children who died under the age of 1 year were based on death certificates, which are required to be completed by either a coroner or medical examiner in all US states, following CDC guidelines. Even though regulations vary by state, deaths due to violence or suspicious circumstances are further investigated and certified by a medical legal officer [6].

Death certificates were reviewed and coded following ICD-10 guidelines by the NCHS. For the purpose of this paper, we grouped reported causes of death into 4 main categories: (1) congenital malformations: ICD-10 codes Q00–Q99; (2) sudden unexpected death in infancy (SUDI): ICD-10 codes V01–Y89 and R00–R99; (3) perinatal conditions: ICD-10 codes P00–P96; and (4) all other causes: all other ICD-10 codes.

The SUDI grouping was chosen intentionally to minimize potential state-level differences in the attribution of unexplained deaths to sudden infant death syndrome (SIDS) versus “other unexplained causes” [7–9]. Some more disaggregated statistics for major causes of deaths (such as SIDS) were also computed as described further below.

### Exclusion criteria

Children born prior to 37 or after 42 weeks of gestation were excluded from this study. All other children born alive in the US between January 1, 2010, and December 31, 2012, including multiple births and children born with malformations (not reported in the NCHS dataset), were analyzed in this study.

### Covariates

In order to assess the extent to which state-level differences in infant mortality rates can be attributed to differences in maternal characteristics, we considered the following variables included in the original data file: mother’s age, educational attainment, smoking behavior, diabetes, chronic hypertension, and eclampsia. We divided maternal age into 5 categories (<20, 20–34, 35–39, 40–44, and >44 years) and used age 20–34 as the reference group in our multivariable analysis. Similarly, we divided maternal educational attainment into 4 categories: less than high school, high school or some college credit without degree, associate or bachelor’s degree, and master’s degree or doctorate. In response to a reviewer request, we also added controls for mother’s race: white, black, American Indian/Alaskan Native, and Asian/Pacific Islander. As for smoking, mothers reported the average number of cigarettes smoked per day during their first, second, and third trimesters. From this we constructed indicators for smoking (number of cigarettes per day > 0) or not for each trimester. We used indicators for previous diagnosis of diabetes, chronic hypertension, and eclampsia as provided in the dataset. All these variables were based on mother’s self-report in the hospital around the time of delivery and were reported on the birth certificate. In addition, we included controls for the following birth characteristics: birth weight category (<1,500, 1,500–1,999, 2,000–2,499, 2,500–2,999, 3,000–3,499, 3,500–3,999, 4,000–4,499, and >4,499 g), multiple birth (1 if singleton, 2 if twin, 3 if triplet, 4 if quadruplet, and 5 if quintuplet or higher), infant sex, and gestational age (indicators for 37 weeks, 38 weeks, 41 weeks, and 42 weeks of gestation) in our empirical models. Further details of all these variables are provided in S1 Table.

### Statistical methods

As a first step, we computed full-term infant mortality rates (FTIMRs) at the state level, and classified all US states into 5 groups: states with excellent FTIMR (FTIMR < 1.25—the European benchmark shown in Fig 1), states with good FTIMR (1.25 ≤ FTIMR < 1.75), states with average FTIMR (1.75 ≤ FTIMR < 2.25), states with fair FTIMR (2.25 ≤ FTIMR < 2.75), and finally states with poor FTIMR (FTIMR ≥ 2.75). The “excellent” group was chosen based on the FTIMRs observed in 6 European countries (Austria, Denmark, Finland, Norway, Sweden, and Switzerland), which ranged between 0.97 and 1.24, with a median FTIMR of 1.11. The remaining groups were defined by sequentially adding 0.5 deaths per 1,000 full-term live births (a 50% increase relative to the European average) to the cutoffs. In a second step, we decomposed mortality differences at the group level by cause of death. Third, we used multivariable regression models to assess the extent to which survival differences across states can be attributed to observable differences in maternal and birth characteristics. To do so, we first ran multivariable logistic models comparing infants born in the states with the highest mortality rates to infants born in the states with the lowest mortality rates. We estimated 3 separate models: a first model, where we did not adjust for any covariates; a second model, where we adjusted for maternal characteristics outlined in the covariates section above; and a third model (proposed by a reviewer), where we adjusted for maternal characteristics and birth characteristics (gestational age, infant sex, birth weight, and multiple birth). Model 2 was estimated to assess the extent to which state-level differences can be attributed to local variation in maternal characteristics such as age, education, race, and health status. Model 3 was estimated to assess the extent to which subsequent mortality differentials were explained by local variation in the prevalence of multiple births as well as differences in birth weight and the distribution of gestational age. In all 3 models, each observation corresponded to a child born full-term in the sample period. To assess the overall contribution of state-level characteristics to variation in FTIMR, we also estimated multilevel logistic models where we nested individual observations within states, and then estimated between-state variance in unconditional models as well as in models conditioning on maternal and birth characteristics. Lastly, we computed hypothetical mortality rates (which we refer to as “counterfactuals”) under the assumptions that (i) all US states achieved the overall FTIMR of the best-performing states (good FTIMR group) and (ii) all US states achieved the specific FTIMRs of the best-performing state in each cause-of-death category.

## Results

**Fig 2:**
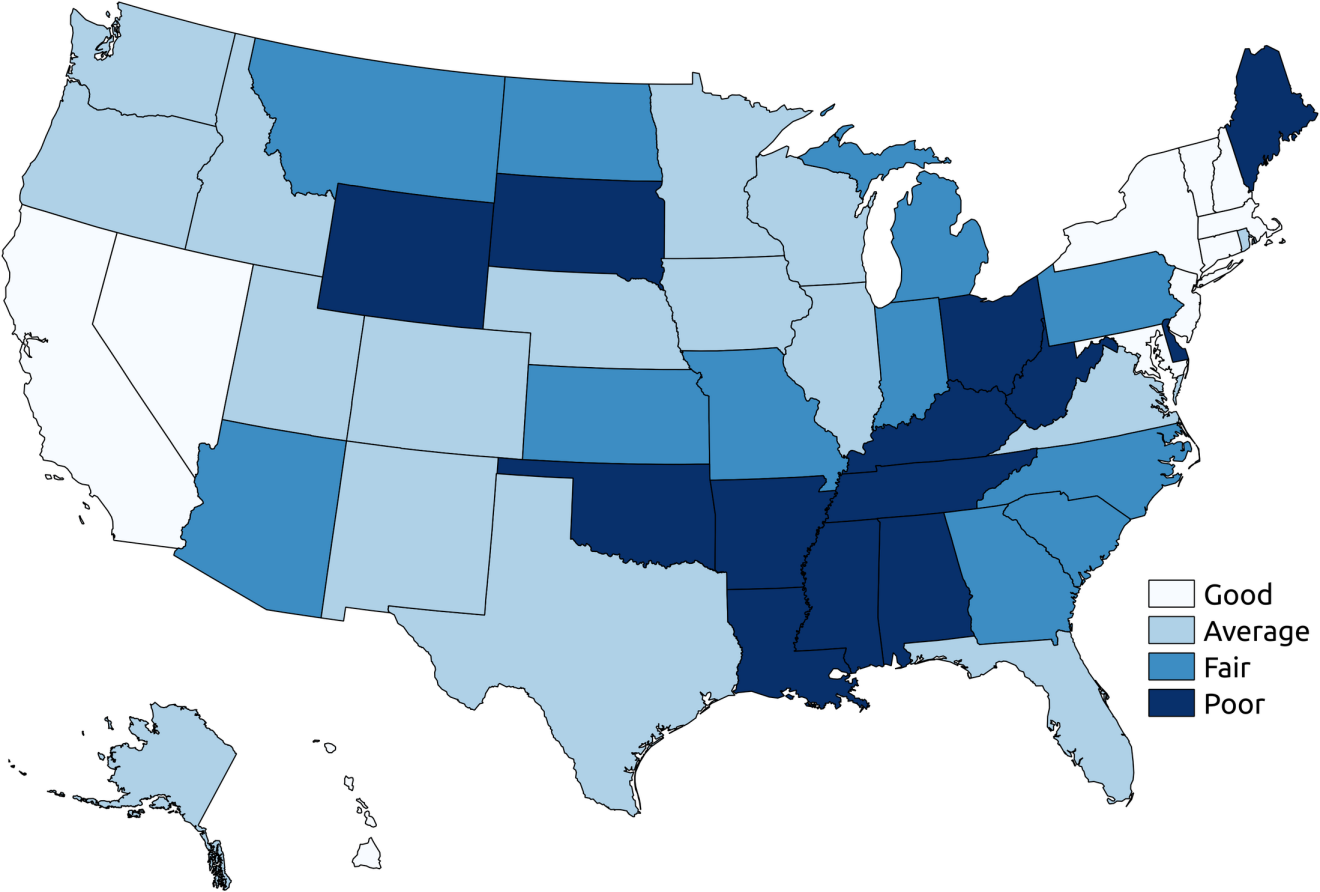
State-level FTIMR classification. The figure shows state level classification: states with good FTIMR (1.25 ≤ FTIMR < 1.75), states with average FTIMR (1.75 ≤ FTIMR < 2.25), states with fair FTIMR (2.25 ≤ FTIMR < 2.75), and states with poor FTIMR (FTIMR ≥ 2.75). All estimates are for full-term infants born in 2010–2012. FTIMR, full-term infant mortality rate.

A total 10,175,481 children born full-term in the US between January 1, 2010, and December 31, 2012, were analyzed. FTIMR was 2.19 (95% CI 2.16, 2.22) per 1,000 full-term live births in the pooled sample. At the state level, estimated FTIMR ranged between 1.29 (95% CI 1.08, 1.53) in Connecticut and 3.77 (95% CI 3.39, 4.19) in Missouri. No state was classified as excellent in terms of their FTIMR; 10 states including Connecticut were classified as good, 17 as average, 11 as fair, and 13 as poor FTIMR (see Fig 2 and S2 Table for details).

**Fig 3:**
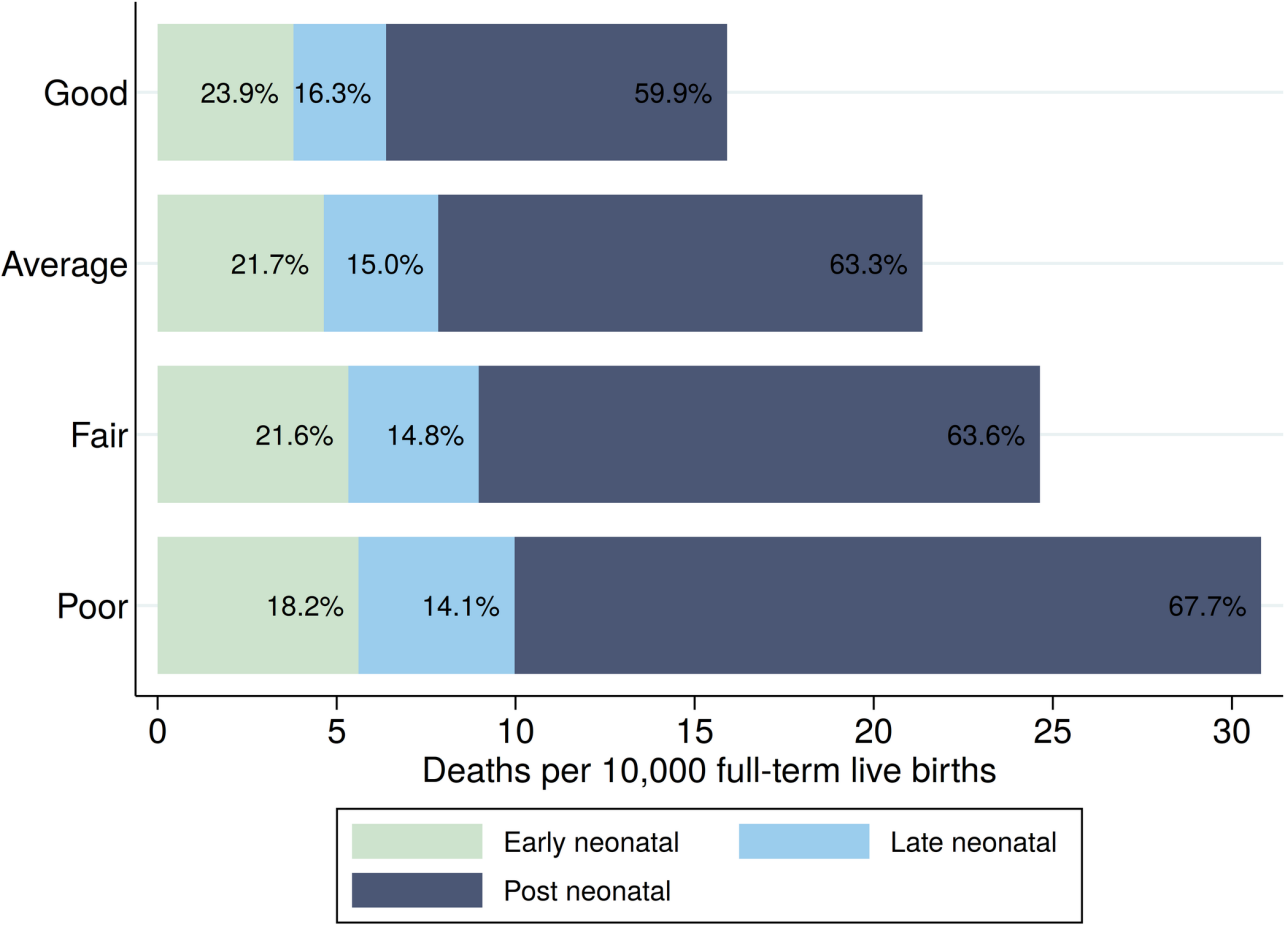
Group-specific mortality by age of death. The figure shows the number of infant deaths per 10,000 full-term births in the US by period and overall mortality group (states grouped on the basis of FTIMR: good, 1.25 ≤ FTIMR < 1.75; average, 1.75 ≤ FTIMR < 2.25; fair, 2.25 ≤ FTIMR < 2.75; and poor, FTIMR ≥ 2.75) for the years 2010 to 2012 as well as the percentage of deaths in each age category. Early neonatal mortality is defined as death in the first 6 days after birth. Late neonatal mortality is defined as deaths between 7 and 27 days after birth, and post-neonatal mortality is defined as death 28 to 364 days after birth. FTIMR, full-term infant mortality rate.

Fig 3 compares early neonatal (death in the first 6 days after birth), late neonatal (death between 7 and 27 days after birth), and post-neonatal (death 28–364 days after birth) mortality rates across mortality groups. While only relatively minor differences were found with respect to early neonatal mortality, large absolute and relative differences were found for the post-neonatal period, with an average of 9.5 (95% CI 9.1, 9.9) deaths per 10,000 full-term births in states classified as having good FTIMR and a mortality rate of 20.9 (95% CI 20.1, 21.6) deaths per 10,000 full-term births in the states classified as having poor FTIMR.

**Fig 4:**
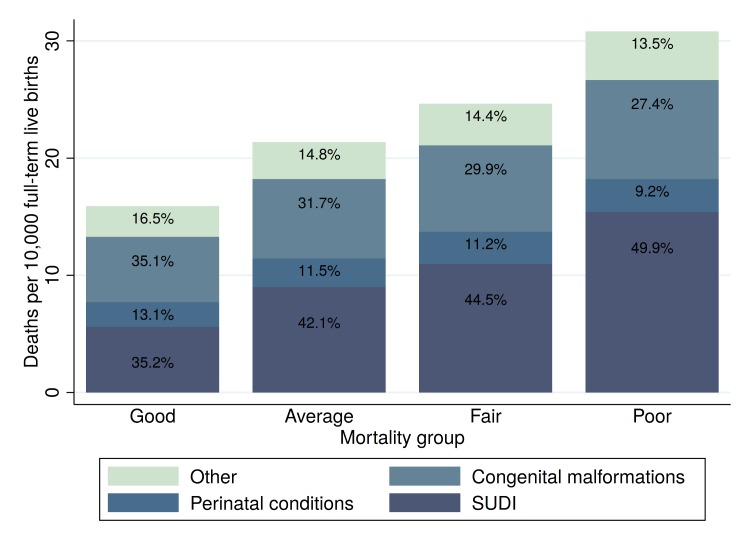
Cause-specific mortality rates. The figure shows the total number of deaths by FTIMR group for the years 2010–2012 as well as the percentage of deaths in each group in the different cause-of-death categories. The following ICD-10 causes of death were included: congenital malformations, Q00–Q99; SUDI, V01–Y89 and R00–R99; perinatal conditions, P00–P96; other, all other ICD-10 codes. Mortality group refers to states grouped on the basis of FTIMR: good (1.25 ≤ FTIMR < 1.75), average (1.75 ≤ FTIMR < 2.25), fair (2.25 ≤ FTIMR < 2.75), and poor (FTIMR ≥ 2.75). FTIMR, full-term infant mortality rate; SUDI, sudden unexpected death in infancy.

Fig 4 summarizes the main causes of full-term infant mortality (FTIM). SUDI accounted for the largest proportion of deaths overall (43%), followed by congenital malformations (31%) and perinatal conditions (11%). The mortality risk due to congenital malformations increased from 5.6 deaths per 10,000 full-term live births in states with FTIMR < 2.75 to 8.4 deaths in states with poor FTIMR. The risk of SUDI was 5.6 in the states classified as having good FTIMR and 15.4 in the states classified as having poor FTIMR. Observed absolute mortality differences between FTIMR groups were smallest for perinatal conditions, with an estimated mortality rate of 2.1 in the states with good FTIMR and an estimated mortality of 2.8 in states with poor FTIMR.

In S1 and S2 Figs, we provide further details on the primary causes of congenital malformations. The 2 most common causes of deaths due to congenital malformation were Edwards syndrome and congenital malformations of the heart, which accounted for 10.9% and 14.6% of congenital malformation deaths, respectively.

In terms of the underlying causes of SUDI, 42.2% of SUDIs were due to SIDS (ICD-10: R95), followed by unknown and ill-defined causes (ICD-10: R99), which accounted for 20.6% of SUDIs, and accidental suffocation and strangulation (ICD-10: W75), which accounted for 16.1% of SUDIs. S3–S7 Figs provide further details on the spatial distribution of cause-specific SUDIs.

S8 Fig summarizes the relative importance of the 4 mortality groups in the neonatal, late neonatal, and post-neonatal periods. Congenital malformations accounted for 58.1% and 43.3% of overall mortality in the early neonatal and late neonatal periods, respectively. Perinatal conditions accounted for 31.7% and 22.8% of mortality in the same periods. In the post-neonatal period—which accounted for the majority of deaths overall (63.5%, as shown in Fig 3)—the large majority (60%) of deaths were due to SUDI.

Table 1 shows estimated OR for the group of states with poor FTIMR compared to the group of states with good FTIMR for the 4 main cause-of-death categories displayed in Fig 4. The table shows unadjusted OR estimates and OR estimates adjusted for the full set of covariates summarized in S1 Table. In unadjusted models, living in a state with poor FTIMR was associated with an increased odds of FTIM due to perinatal conditions of 35% (OR 1.35, 95% CI 1.17, 1.56) as well as an increased odds of death due to congenital malformations of 51% (1.51, 95% CI 1.24, 1.85). Risk differentials were largest for SUDI, with an estimated OR of 2.75 (95% CI 2.46, 3.07). When we adjusted for maternal age, education, race, and measures of health status, estimated risk differentials declined for all risk factors, with the largest declines for SUDI, where estimated OR fell from 2.75 in unadjusted models to 1.70 in models adjusting for both maternal and birth characteristics. In general, differences between models 2 (adjusting for maternal characteristics only) and 3 (adjusting for maternal characteristics and birth characteristics) were small and not statistically significant.

**Table 1:** Relative odds of cause-specific full-term infant mortality in states with poor FTIMR relative to states with good FTIMR.

Cause of death	Unadjusted odds		Adjusted odds			
	Model 1		Model 2		Model 3	
	Odds ratio	95% CI	Odds ratio	95% CI	Odds ratio	95% CI
Congenital malformations	1.51**	1.24, 1.85	1.43**	1.28, 1.59	1.37**	1.19, 1.58
Perinatal conditions	1.35**	1.17, 1.56	1.19	1.01, 1.42	1.16	0.97, 1.38
SUDI	2.75**	2.46, 3.07	1.73**	1.51, 1.98	1.70**	1.48, 1.94
Other causes	1.58**	1.37, 1.82	1.40**	1.16, 1.67	1.37**	1.15, 1.64

Table shows relative odds of cause-specific mortality in states with overall poor FTIMR compared to states classified with good FTIMR. The states classified as having good FTIMR include CA, CT, HI, MA, MD, NH, NJ, NV, NY, and VT (total births 2,885,191; total deaths 4,589), and the states classified as having poor FTIMR include AL, AR, DE, KY, LA, ME, MS, OH, OK, SD, TN, WY, and WV (total births 1,476,604; total deaths 4,551). Model 2 adjusts for maternal characteristics including mother’s age, education, race, and health status (smoking behavior, diabetes, chronic hypertension, and eclampsia). Model 3 adjusts for maternal characteristics and birth characteristics including gestational age, infant sex, birth weight, and multiple birth.

**p < 0.01.

FTIMR, full-term infant mortality rate; SUDI, sudden unexpected death in infancy.

In Table 2, we show estimated state variability in mortality outcomes based on multilevel logistic models. State-level variation was highest for SUDI (estimated state-level variance 0.118, 95% CI 0.068, 0.168) and congenital malformations (0.061, 95% CI 0.028, 0.095). These state-level differences were reduced substantially for all causes when we controlled for differences in maternal and birth characteristics, with particularly large reductions for SUDI, where estimated state variability dropped to 0.034 (95% CI 0.014, 0.054) when both maternal and birth characteristics were included in the model.

**Table 2:** Variation (on logit scale) in cause-specific mortality between US states estimated using the random intercept logistic model.

Model	Variance (95% CI) by cause of death			
	Congenital malformations	Perinatal conditions	SUDI	Other causes
Model 1	0.061** (0.028, 0.095)	0.021 (0.002, 0.039)	0.118** (0.068, 0.168)	0.033* (0.012, 0.054)
Model 2	0.032* (0.011, 0.054)	0.012 (0.000, 0.026)	0.035** (0.014, 0.056)	0.025 (0.003, 0.039)
Model 3	0.036* (0.013, 0.058)	0.012 (0.000, 0.020)	0.034** (0.014, 0.054)	0.025 (0.003, 0.039)

Estimates show state-level variation in mortality outcomes. Variances as well as 95% confidence intervals estimated using multivariable logistic model, where individuals (level 1) are nested into states (level 2). The results of the fully specified model are displayed in S4 Table.

*p < 0.05

**p < 0.01.

SUDI, sudden unexpected death in infancy.

Table 3 shows estimated annual FTIM for our 2 hypothetical scenarios. Under the assumption that all states would achieve the survival outcomes of the 10 states with the lowest mortality outcomes overall (good FTIMR group), infant mortality would decline by an estimated 2,023 (95% CI 1,717, 2,329) deaths each year. Under the more ambitious counterfactual that all states could achieve the cause-specific mortality rates of the best-performing state in each cause-of-death category, infant mortality among full-term births would be reduced by 4,003 deaths (95% CI 2,284, 5,587) each year. Under both hypothetical scenarios, only about 10% of the potential improvements were related to perinatal conditions or other causes. More than 75% of the excess burden of mortality in both scenarios was due to congenital malformations and SUDI.

**Table 3:** Estimated preventable deaths among full-term births.

Cause of death	Actual number of deaths 2010–2012	Counterfactual scenario			
		Mortality of good FTIMR group		Best US state	
		Predicted deaths (95% CI)	Mortality reduction	Predicted deaths (95% CI)	Mortality reduction
Congenital malformations	2,308	1,897 (1,805, 1,990)	411	683 (0, 1,456)	1,625
Perinatal conditions	839	711 (654, 767)	128	340 (0, 724)	499
SUDI	3,187	1,906 (1,813, 1,998)	1,281	1,752 (1,458, 2,046)	1,435
Other	1,098	894 (831, 958)	203	654 (387, 921)	444
All	7,431	5,408 (5,102, 5,714)	2,023	3,428 (1,844, 5,147)	4,003

Based on an estimated 3.4 million full-term live births per year. The best state estimates are from Vermont (congenital malformations, 2.01 deaths per 10,000 full-term births, 95% CI 0, 4.28), Rhode Island (perinatal conditions, 1.00 deaths per 10,000 full-term births, 95% CI 0, 2.13), New Jersey (SUDI, 5.15 deaths per 10,000 full-term births, 95% CI 4.29, 6.02), and Oregon (other causes, 1.92 deaths per 10,000 full-term births, 95% CI 1.14, 2.71). Good FTIMR group refers to states with 1.25 ≤ FTIMR < 1.75.

FTIMR, full-term infant mortality rate; SUDI, sudden unexpected death in infancy.

## Discussion

The results presented in this paper show a large gap in the survival probabilities of full-term infants born in the US compared to European countries with low under-5 mortality rates. Pooling all available data between 2010 and 2012, we found that no single US state or territory achieved the full-term survival rates currently reported in leading European countries, with children born full-term in the 10 best-performing states facing about 50% higher risks of infant mortality, and children born in states with poor FTIMR facing almost 3 times the infant mortality risk of European countries with low infant mortality rates.

Given that survival rates among preterm infants in the US were found to be very similar to those of the same European countries (as illustrated in Fig 1), clinical care during or immediately after delivery likely does not explain much of the mortality gap observed. In the sample analyzed, perinatal conditions—where healthcare quality likely matters most—accounted only for about 11% of total infant mortality among full-term births. In terms of the big picture, the high burden of FTIM in the US seemed to be mostly due to SUDI and congenital malformations, which accounted for 42.9% and 31.1% of the total infant mortality burden among full-term children, respectively, and for almost 80% of excess deaths in our counterfactual analysis. From a policy perspective, deaths due to malformations are quite different from deaths classified as SUDI. Malformations are in practice hard, if not impossible, to prevent; in most cases, the only way to “prevent” malformation-related infant mortality is to increase screening and early termination. In terms of the overall magnitude, we found malformation-specific FTIMRs of less than 3 per 10,000 live births in some states, such as Vermont and New Jersey, and rates 3 times higher in quite a few states in the Mississippi delta and surrounding states (see S2 Fig for details). Globally, WHO estimates suggest that 330,000 children die annually during the neonatal period due to congenital malformations [10,11], which corresponds to a risk of approximately 2.5 deaths per 10,000. Taking these global estimates as a benchmark suggests that children in the US face about 3 times the risk of death due to malformation in other countries. In practice, the extent to which these differences reflect differences in screening and termination policies rather than differences in medical care across states and countries is not clear; further research investigating the reach and effectiveness of early screening programs across countries and states will be needed to better understand these current gaps.

With respect to actual health improvements, the area with the most obvious and ample room for increasing the chances of child survival is SUDIs. Given that the attribution of deaths to SIDS versus “other unexplained causes” was not obvious in many cases [7,8], we mostly focused on the larger SUDI category in this paper. More than 3,000 infants died in the US each year between 2010 and 2012 due to causes that were—as the name suggests—not expected under normal conditions. This is perhaps most immediately obvious when it comes to accidental suffocation or strangulation in bed. Over 600 infants die in the US each year due to suffocation in bed; new strategies to convey optimal sleeping arrangements to parents will need to be developed and tested to prevent these deaths.

SUDI mortality in the best-performing states of the US (California and New York) was less than 6 deaths per 10,000 births; rates were more than twice as high (>12) in 12 states, including Ohio, South Dakota, and Tennessee. A large fraction of these deaths were attributed to SIDS, which has previously been estimated to cause 6.4 deaths per 10,000 births [12]. Our results suggested SIDS incidence rates as low as 1.27 and 1.32 per 10,000 full-term live births in Nevada and New Mexico and as high as 13.33 and 8.75 in Arkansas and Mississippi. Evidence from European studies suggests that a large majority of SIDS deaths could historically be attributed to prone sleeping and maternal drug consumption [13]. Through active public health programs, the incidence of SIDS was lowered by 75% in Sweden [14] and Scotland [15]; general compliance with sleeping recommendations continues to be a challenge in the US, particularly among women with low socioeconomic status [16]. Empirically, a large proportion of the state-level differences in mortality due to both SIDS and the broader SUDI category could be attributed to state-level differences in maternal age and maternal education. As shown in the more detailed regression results in S4 Table, maternal characteristics were highly predictive of these mortality outcomes. We found that compared to children born to mothers with incomplete high school education, children of highly educated mothers (those with master’s degree or doctorate) had 74% lower odds of SUDI, and that the risk of SUDI almost linearly declined with maternal age (conditional on all other factors). This suggests that mortality in this category is strongly influenced by maternal behavior and the early home environment, both of which should at least in principle be modifiable through targeted information and behavioral change interventions.

Our analysis is not without limitations. First, we have relatively little information on children’s home environments, and thus cannot directly identify what is happening at children’s homes or compare underlying risk factors. Second, it is possible that state-level estimates that we present may be biased if people move before or after birth. Empirically, for 97% of the observations, state of birth is the same as state of residence, which means that these biases should be small if they exist. Third, as mentioned above, we do not have information on termination rates at the state level, which are likely to (at least partially) explain differences in birth outcomes observed. According to the latest estimates available, approximately 700,000 legally induced abortions occurred in 2012 in the US [17], which corresponds to about 20% of the annual sample analyzed in this study. While it seems likely that infant mortality rates would be higher without these terminations, our data do not allow us to directly quantify these differences. Last, it seems likely that some of the less common causes of death (particularly in the ICD-10 R and W categories) are miscoded or coded differentially across states. To reduce this type of measurement error, we grouped all SUDIs together for most of our analyses.

### Conclusion

More than 7,000 children born alive at full-term in the US each year die within their first year of life. The results presented in this paper suggest that a substantial proportion of these deaths are preventable, with particularly large improvements possible for SUDI.

## Supporting information

S1 Fig Primary causes of death due to malformation. The figure shows the FTIMR burden for the 7 most common causes of death due to malformation by mortality group among full-term infants born in 2010–2012.
(TIF)

S2 Fig Spatial distribution of full-term infant mortality due to malformations. The figure shows the number of infant deaths per 10,000 full-term births due to malformations among full-term infants born in 2010–2012.
(TIF)

S3 Fig Primary causes of deaths due to SUDI. The figure shows the FTIMR burden for the most common causes of death classified as SUDI by mortality group among full-term infants born in 2010–2012.
(TIF)

S4 Fig Infant deaths per 10,000 full-term live births classified as SUDI. The figure shows the number of infant deaths per 10,000 full-term infants born in 2010–2012. Estimates include all deaths filed under ICD-10 codes V01–Y89 and R00–R99.
(TIF)

S5 Fig Infant deaths per 10,000 full-term live births due to violence or assault. The figure shows the number of infant deaths per 10,000 full-term infants born in 2010–2012. Estimates include all deaths filed under ICD-10 codes Y079 (unspecified perpetrator of maltreatment and neglect) and Y09 (assault by unspecified means).
(TIF)

S6 Fig Infant deaths per 10,000 full-term live births due to suffocation. The figure shows the number of deaths per 10,000 full-term births in 2010–2012 due to suffocation. Estimates include all deaths filed under ICD-10 codes W75 (accidental suffocation and strangulation in bed) and W84 (unspecified threat to breathing).
(TIF)

S7 Fig Deaths per 10,000 full-term live births across states due to SIDS. The figure shows the number of deaths per 10,000 full-term births in 2010–2012 due to SIDS. Estimates include all deaths filed under ICD-10 code R95 (sudden infant death syndrome).
(TIF)

S8 Fig Percentage of deaths in early, late, and post-neonatal periods due to specific causes. The figure shows the percentage of deaths occurring due to each cause of death in the early neonatal (1), late neonatal (2), and post-neonatal periods (3) in the years 2010–2012. Early neonatal mortality is defined as death in the first 6 days after birth. Late neonatal mortality is defined as deaths between 7 and 27 days after birth, and post-neonatal mortality is defined as deaths 28 to 364 days after birth.
(TIF)

S1 Table Sample characteristics. (DOCX)

S2 Table State-specific mortality rates. (DOCX)

S3 Table Cause-specific mortality distribution by mortality group. (DOCX)

S4 Table Estimates from random intercept logistic model (odds ratio). (DOCX)

S5 Table Estimates from a logistic model using mortality groups (odds ratio). (DOCX)

## Abbreviations

CDC: US Centers for Disease Control and Prevention
FTIM: full-term infant mortality
FTIMR: full-term infant mortality rate
NCHS: National Center for Health Statistics
OR: odds ratio
SIDS: sudden infant death syndrome
SUDI: sudden unexpected death in infancy
