# Supplementary figures and images for: Causes of death and infant mortality rates among full-term births in the United States between 2010 and 2012: An observational study
Source: PLoS Med. 2018 Mar 20;15(3):e1002531. doi: 10.1371/journal.pmed.1002531 (PMC5860700; doi:10.1371/journal.pmed.1002531)

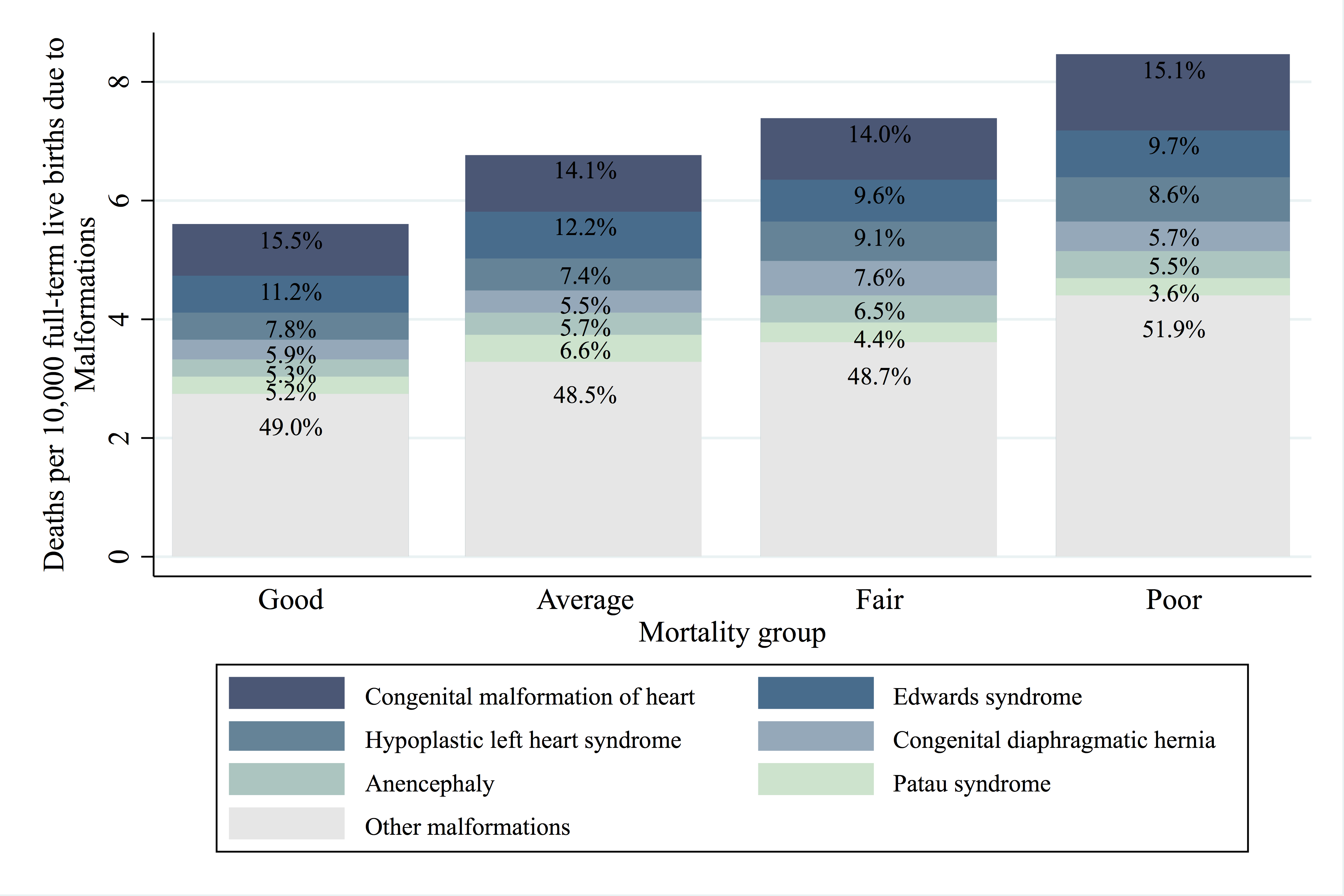

Supplement: S1 Fig — The figure shows the FTIMR burden for the 7 most common causes of death due to malformation by mortality group among full-term infants born in 2010–2012. (TIF) [file pmed.1002531.s001.tif]

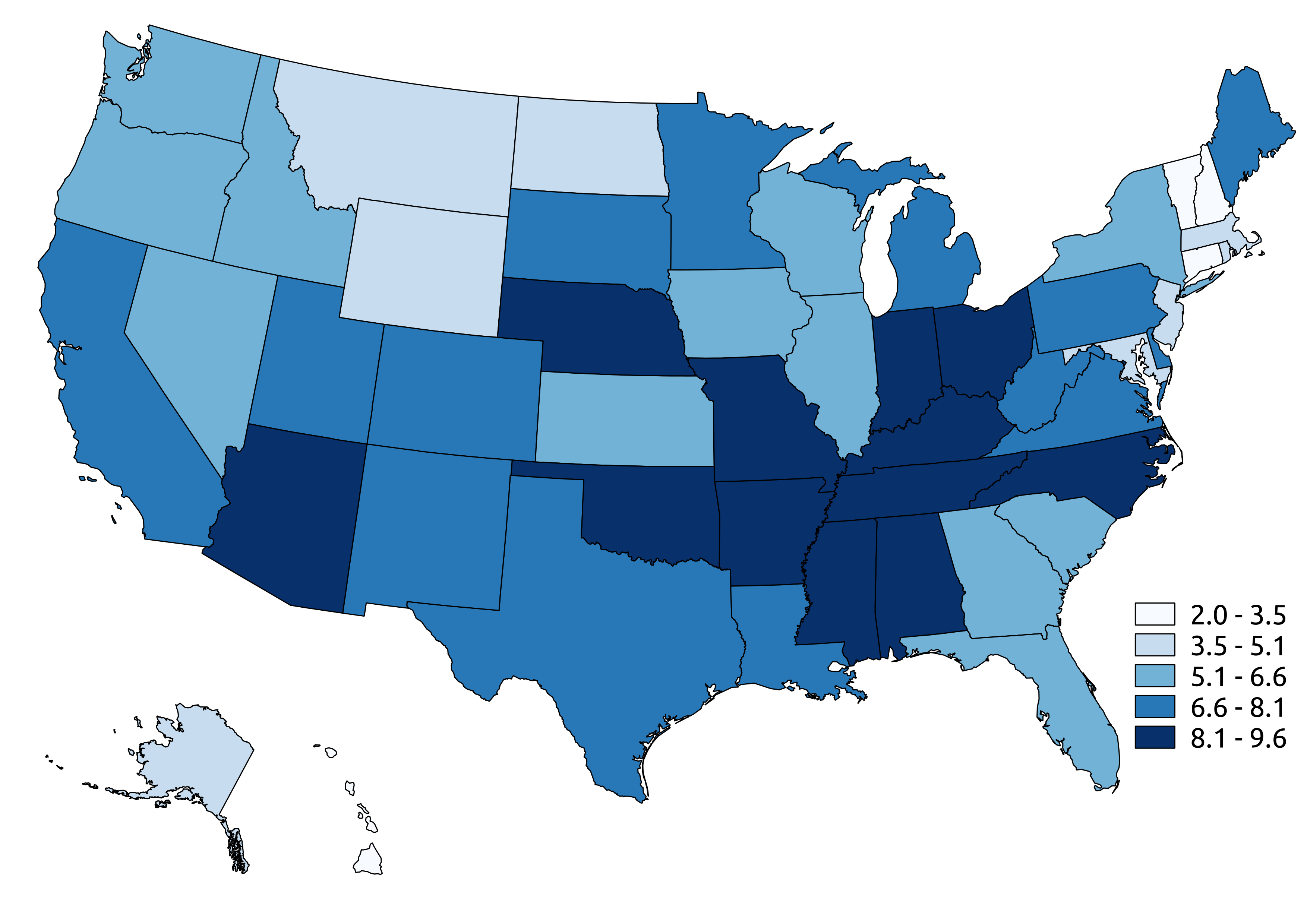

Supplement: S2 Fig — The figure shows the number of infant deaths per 10,000 full-term births due to malformations among full-term infants born in 2010–2012. (TIF) [file pmed.1002531.s002.tif]

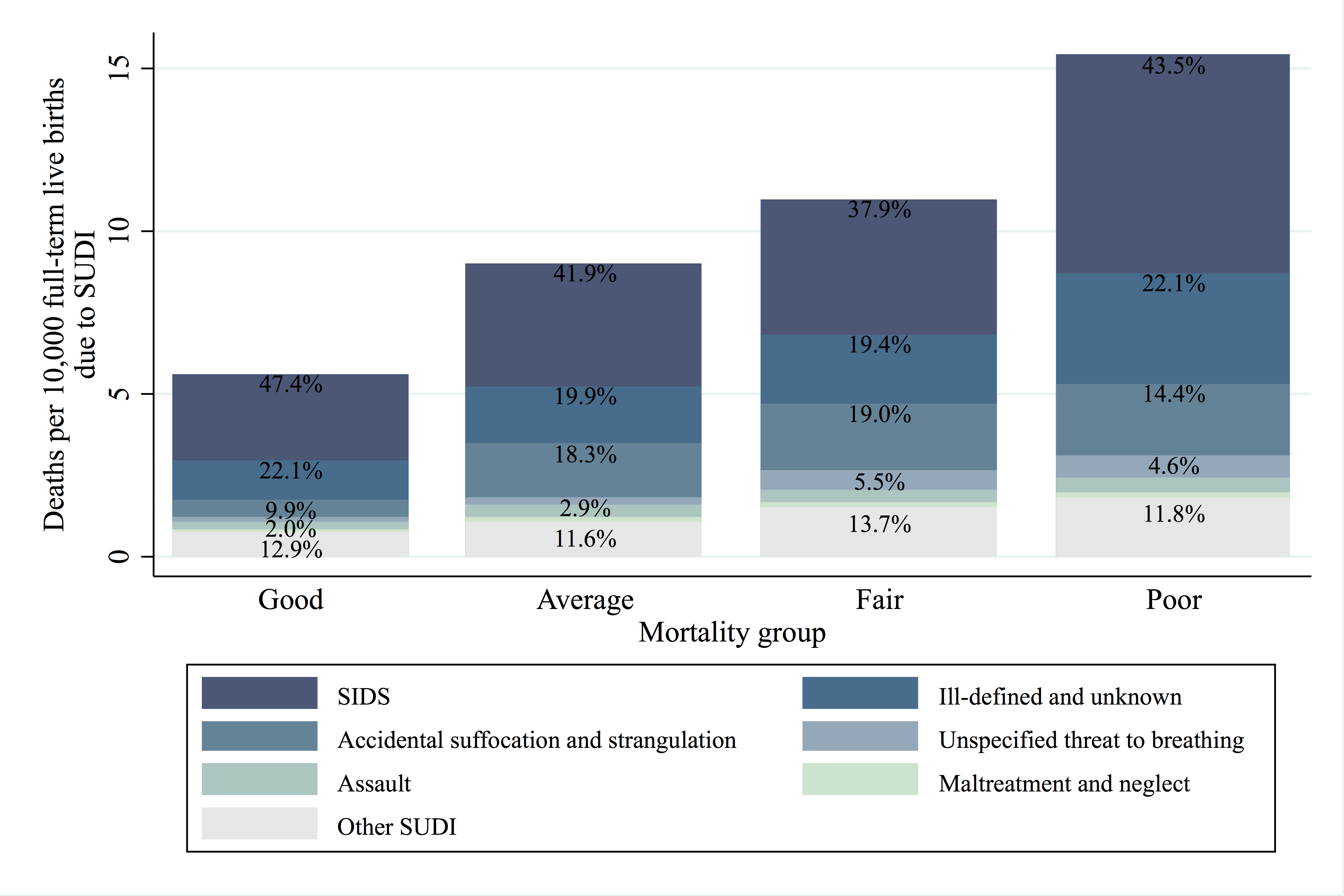

Supplement: S3 Fig — The figure shows the FTIMR burden for the most common causes of death classified as SUDI by mortality group among full-term infants born in 2010–2012. (TIF) [file pmed.1002531.s003.tif]

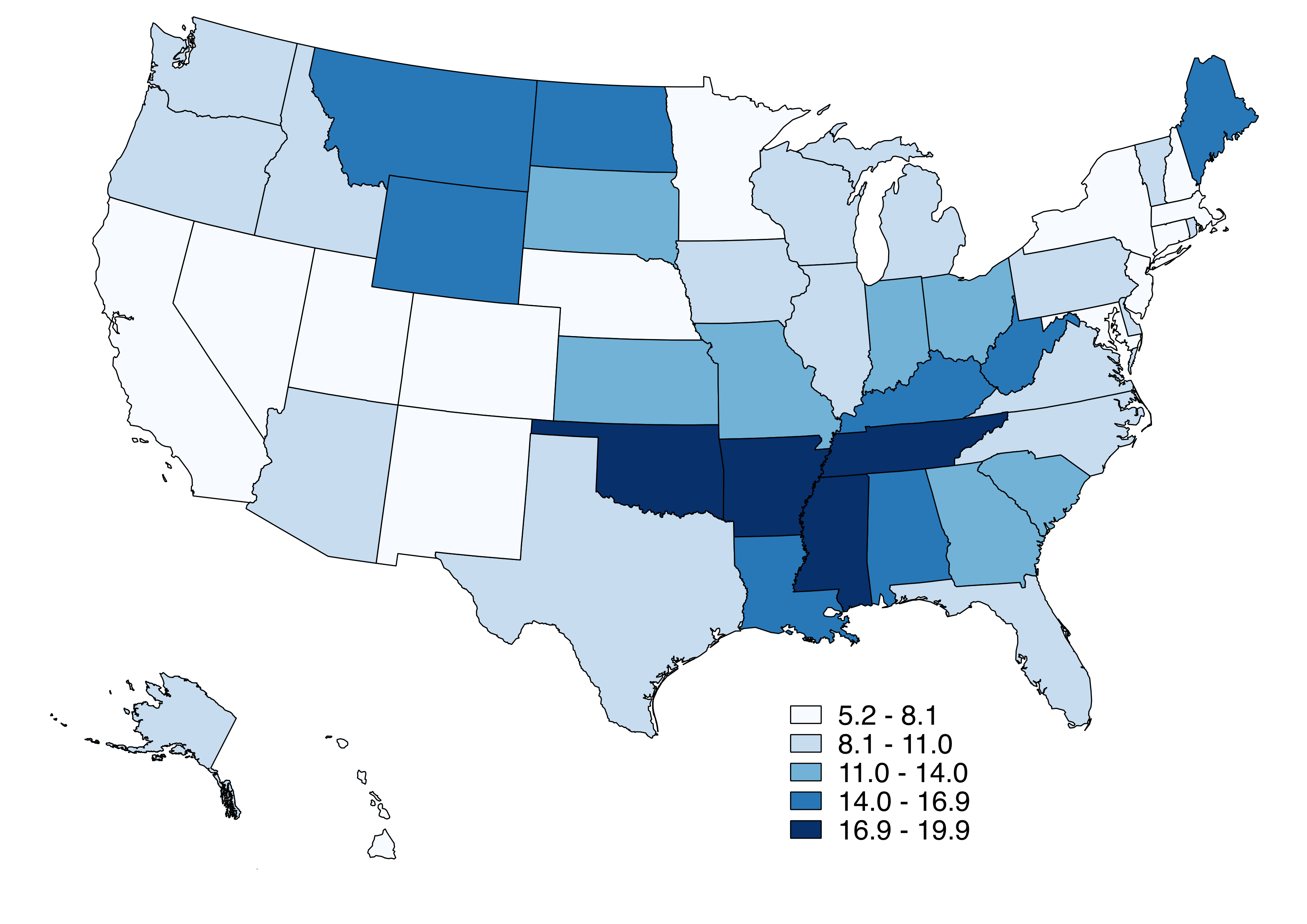

Supplement: S4 Fig — The figure shows the number of infant deaths per 10,000 full-term infants born in 2010–2012. Estimates include all deaths filed under ICD-10 codes V01–Y89 and R00–R99. (TIF) [file pmed.1002531.s004.tif]

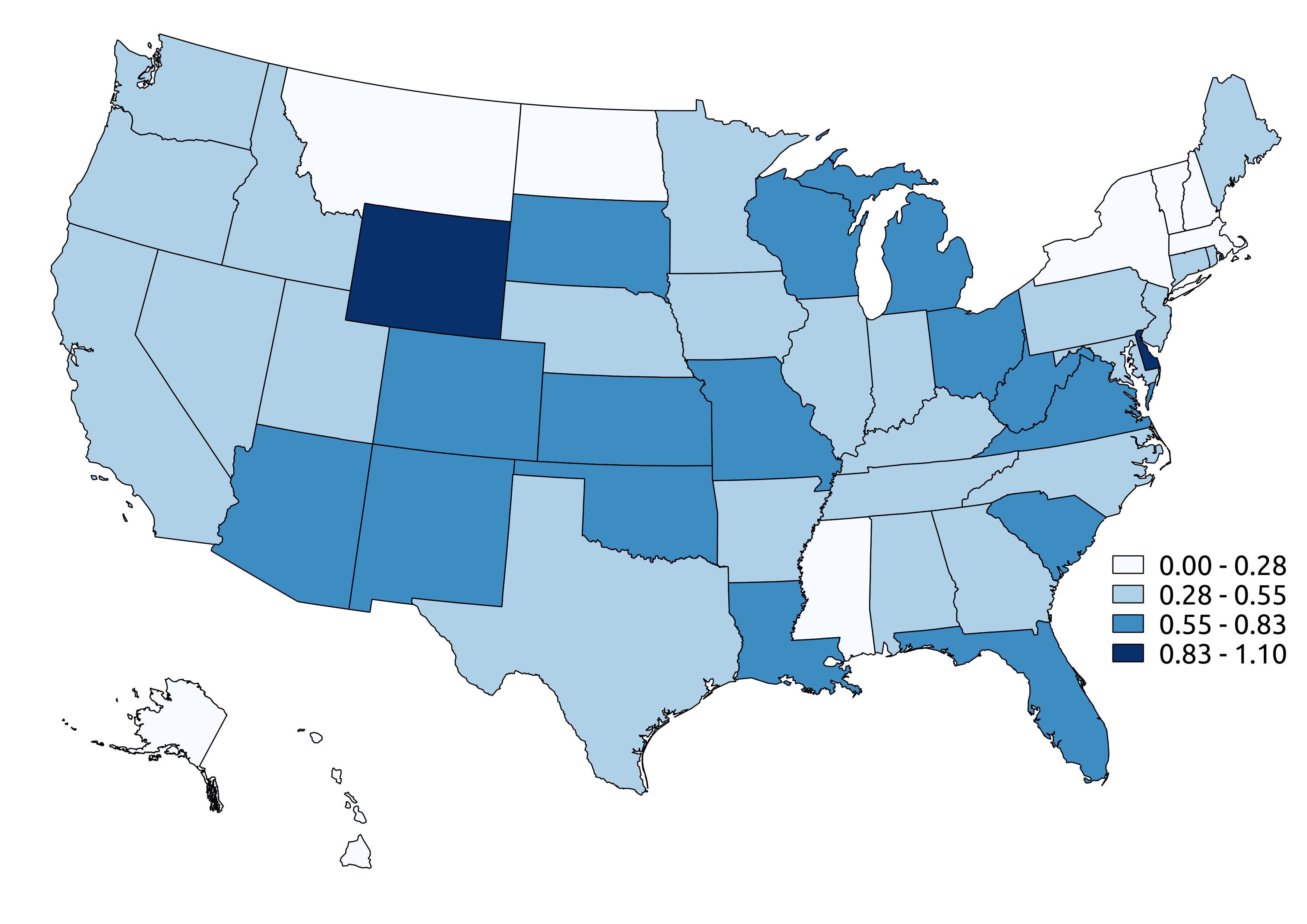

Supplement: S5 Fig — The figure shows the number of infant deaths per 10,000 full-term infants born in 2010–2012. Estimates include all deaths filed under ICD-10 codes Y079 (unspecified perpetrator of maltreatment and neglect) and Y09 (assault by unspecified means). (TIF) [file pmed.1002531.s005.tif]

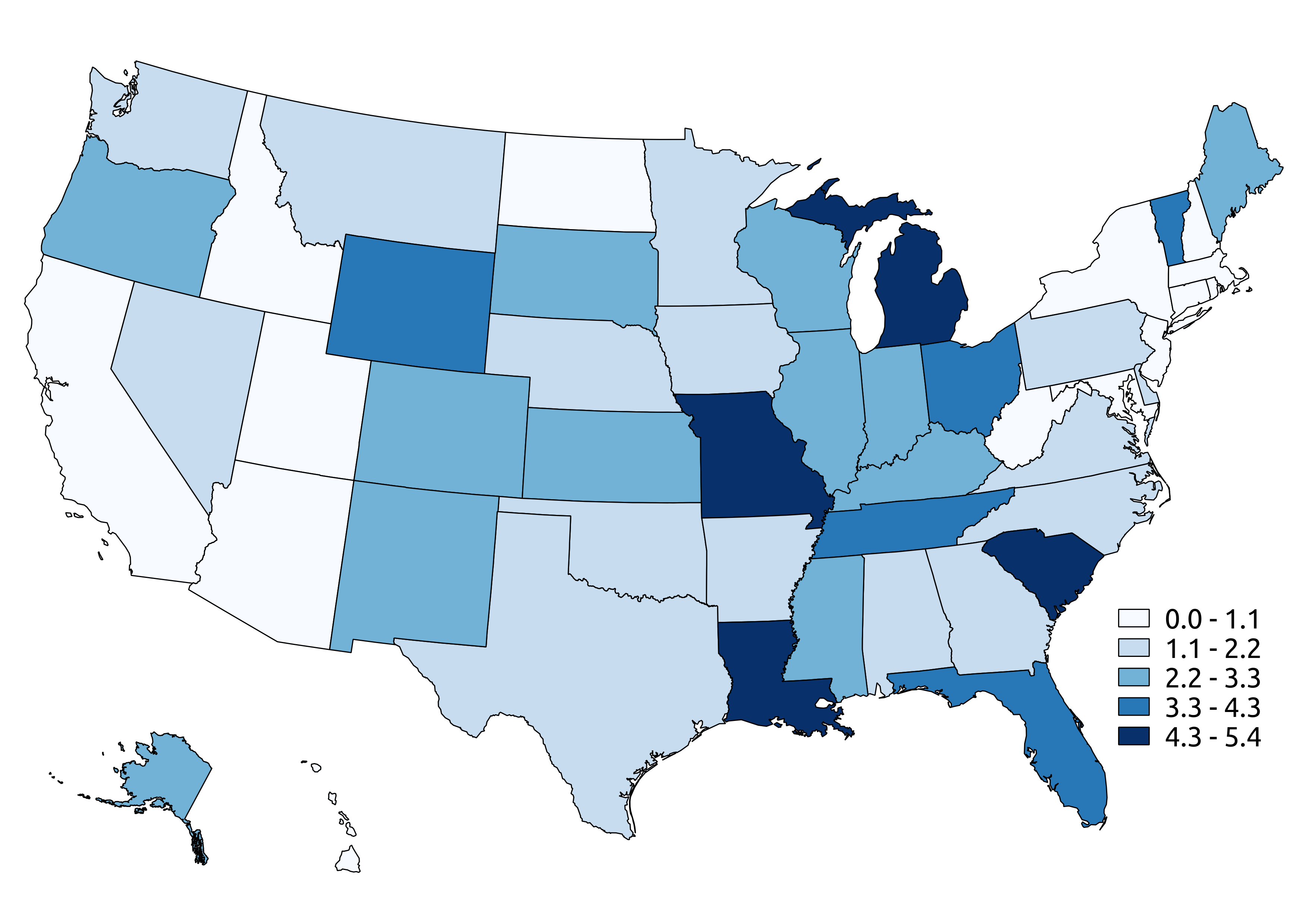

Supplement: S6 Fig — The figure shows the number of deaths per 10,000 full-term births in 2010–2012 due to suffocation. Estimates include all deaths filed under ICD-10 codes W75 (accidental suffocation and strangulation in bed) and W84 (unspecified threat to breathing). (TIF) [file pmed.1002531.s006.tif]

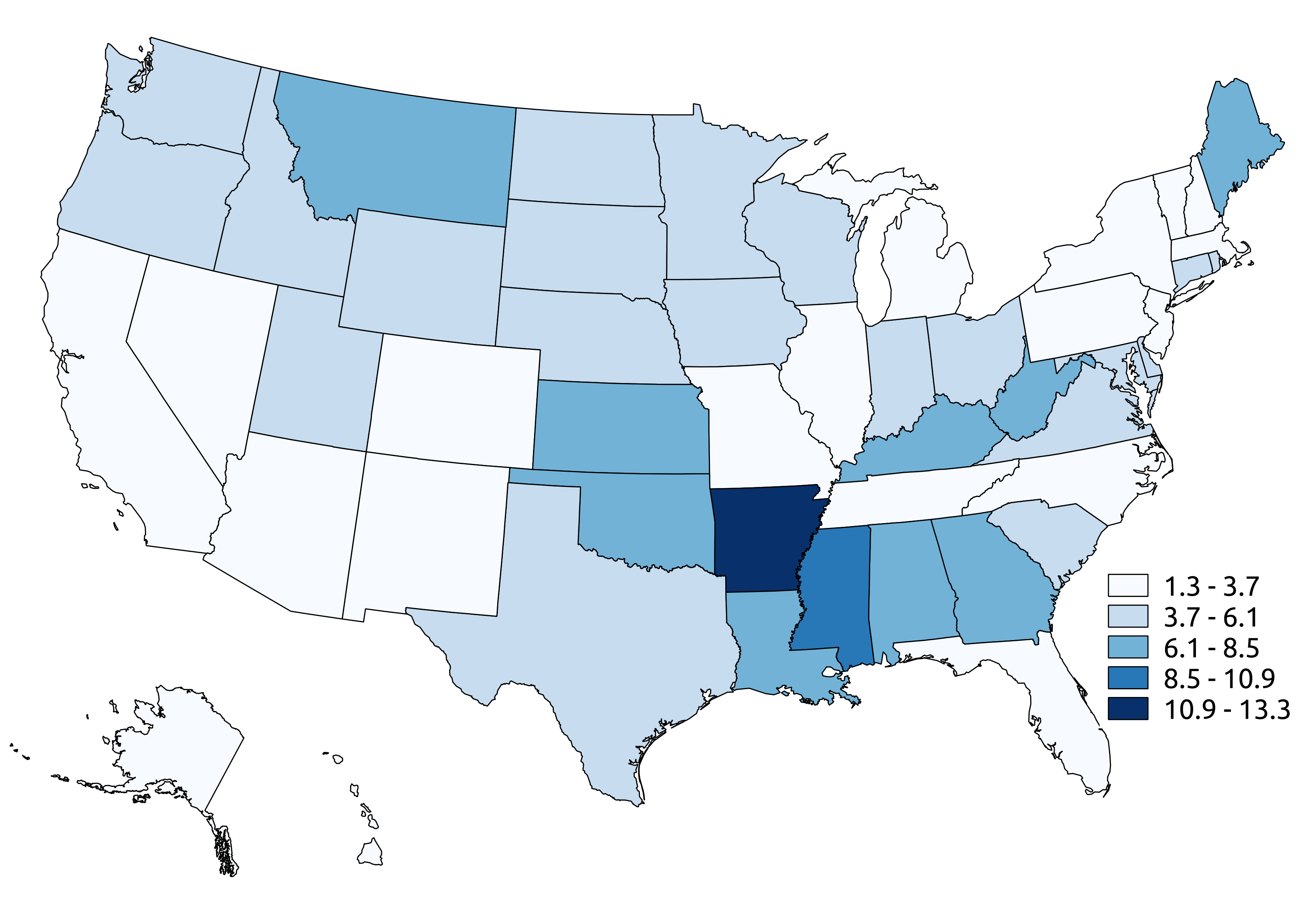

Supplement: S7 Fig — The figure shows the number of deaths per 10,000 full-term births in 2010–2012 due to SIDS. Estimates include all deaths filed under ICD-10 code R95 (sudden infant death syndrome). (TIF) [file pmed.1002531.s007.tif]

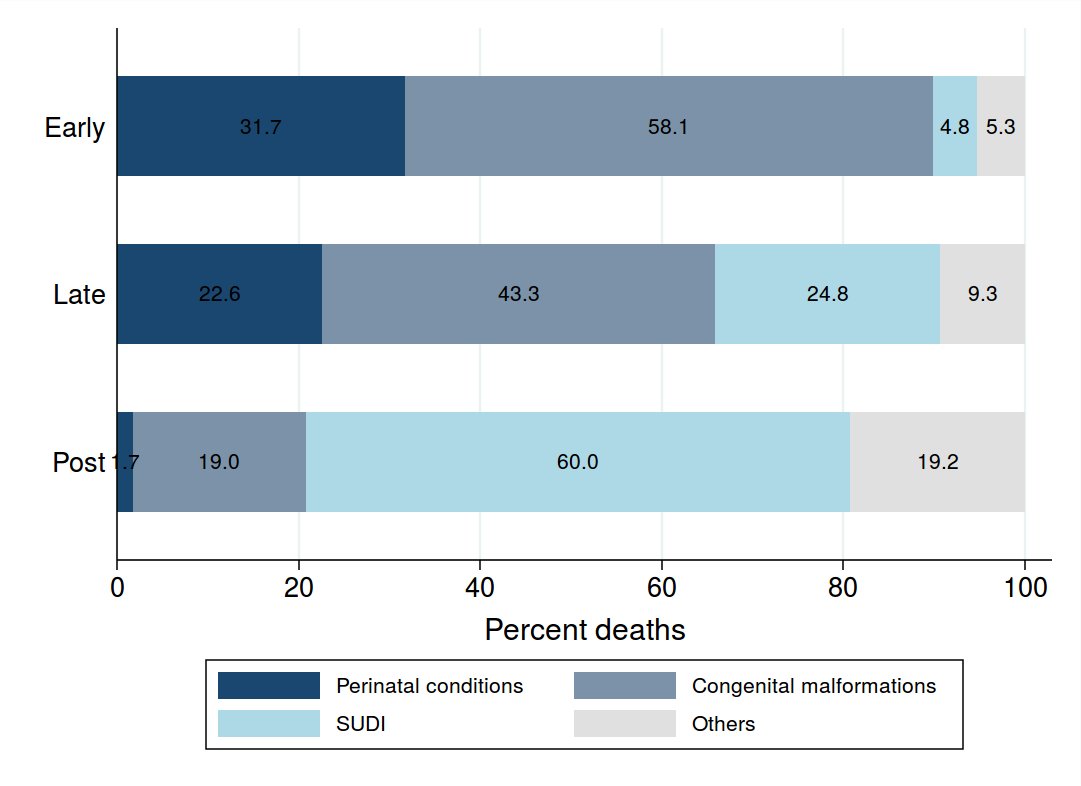

Supplement: S8 Fig — The figure shows the percentage of deaths occurring due to each cause of death in the early neonatal (1), late neonatal (2), and post-neonatal periods (3) in the years 2010–2012. Early neonatal mortality is defined as death in the first 6 days after birth. Late neonatal mortality is defined as deaths between 7 and 27 days after birth, and post-neonatal mortality is defined as deaths 28 to 364 days after birth. (TIF) [file pmed.1002531.s008.tif]
