# Supplementary material for: Causes of death and infant mortality rates among full-term births in the United States between 2010 and 2012: An observational study
Source: PLoS Med. 2018 Mar 20;15(3):e1002531. doi: 10.1371/journal.pmed.1002531 (PMC5860700; doi:10.1371/journal.pmed.1002531)
Supplement: S1 Table — (DOCX) [file pmed.1002531.s009.docx]

|  | Good | Average | Fair | Poor | Total |
| --- | --- | --- | --- | --- | --- |
| Number of full-term live births | 2,885,191 | 3,575,864 | 2,237,822 | 1,476,604 | 10,175,481 |
|  |  |  |  |  |  |
| Cause specific deaths (N) |  |  |  |  |  |
|  |  |  |  |  |  |
| SUDI | 1617 | 3217 | 2454 | 2272 | 9560 |
|  |  |  |  |  |  |
| Congenital malformations | 1610 | 2418 | 1649 | 1247 | 6924 |
|  |  |  |  |  |  |
| Perinatal conditions | 603 | 877 | 618 | 418 | 2516 |
|  |  |  |  |  |  |
| Others | 759 | 1127 | 793 | 614 | 3293 |
|  |  |  |  |  |  |
| Total full-term deaths | 4589 | 7639 | 5514 | 4551 | 22293 |
|  |  |  |  |  |  |
| Mothers characteristics |  |  |  |  |  |
|  |  |  |  |  |  |
| Age Group (N %) |  |  |  |  |  |
| Age < 20 | 188847 (6.55 %) | 296208 (8.28 %) | 199432 (8.91 %) | 155323 (10.52 %) | 839810 (8.25 %) |
|  |  |  |  |  |  |
| Age 20-34 | 2147292 (74.42 %) | 2778098 (77.69 %) | 1759575 (78.63 %) | 1175067 (79.58 %) | 7860032 (77.24 %) |
|  |  |  |  |  |  |
| Age 35-39 | 435038 (15.08 %) | 407686 (11.40 %) | 227826 (10.18 %) | 120575 (8.17 %) | 1191125 (11.71 %) |
|  |  |  |  |  |  |
| Age 40-44 | 106687 (3.70 %) | 88652 (2.48 %) | 48275  (2.16 %) | 24306 (1.65 %) | 267920 (2.63 %) |
|  |  |  |  |  |  |
| Age > 44 | 7327  (0.25 %) | 5220  (0.15 %) | 2714  (0.12 %) | 1333  (0.09 %) | 16594  (0.16 %) |
|  |  |  |  |  |  |
| Education (N %) |  |  |  |  |  |
| < High School | 456663  (15.83 %) | 555858 (15.54 %) | 310531 (13.88 %) | 170715 (11.56 %) | 1493767 (14.68 %) |
|  |  |  |  |  |  |
| High School/College Credit | 1544982 (53.55 %) | 1859683 (52.01 %) | 1219180 (54.48 %) | 965841 (65.41 %) | 5589686 (54.93 %) |
|  |  |  |  |  |  |
| Associate/Bachelor's Degree | 587772 (20.37 %) | 864037 (24.16 %) | 518811 (23.18 %) | 249925 (16.93 %) | 2220545 (21.82 %) |
|  |  |  |  |  |  |
| Master's Degree/Doctorate | 295774  (10.25 %) | 296286 (8.29 %) | 189300  (8.46 %) | 90123  (6.10 %) | 871483  (8.56 %) |
|  |  |  |  |  |  |
| Mothers Health (N %) |  |  |  |  |  |
| Diabetes | 5614679 (194.83 %) | 6940071 (194.74 %) | 4330212 (194.85 %) | 2865695 (194.84 %) | 1.98e+07 (194.81 %) |
|  |  |  |  |  |  |
| Chronic Hypertension | 5735594 (199.03 %) | 7087902 (198.89 %) | 4414997 (198.67 %) | 2914888 (198.18 %) | 2.02e+07 (198.78 %) |
|  |  |  |  |  |  |
| Eclampsia | 5758952 (199.84 %) | 7122404 (199.86 %) | 4440687 (199.83 %) | 2938002 (199.75 %) | 2.03e+07 (199.83 %) |
|  |  |  |  |  |  |
| Cigarettes 1st trimester | 95114  (3.99 %) | 204656 (6.86 %) | 185320 (13.35 %) | 158085 (15.67 %) | 643175  (8.28 %) |
|  |  |  |  |  |  |
| Cigarettes 2nd trimester | 75994  (3.19 %) | 175658 (5.89 %) | 158419 (11.42 %) | 139239 (13.80 %) | 549310  (7.08 %) |
|  |  |  |  |  |  |
| Cigarettes 3rd trimester | 71880  (3.01 %) | 167033 (5.60 %) | 152669 (11.01 %) | 133935 (13.28 %) | 525517  (6.77 %) |
|  |  |  |  |  |  |
| Gestational age (N %) |  |  |  |  |  |
| 37 weeks | 268651  (9.31 %) | 374730 (10.48 %) | 228877 (10.23 %) | 170211 (11.53 %) | 1042469 (10.24 %) |
|  |  |  |  |  |  |
| 38 weeks | 550827  (19.09 %) | 731615 (20.46 %) | 441048 (19.71 %) | 321455 (21.77 %) | 2044945 (20.10 %) |
|  |  |  |  |  |  |
| 39 weeks | 955096  (33.10 %) | 1215495 (33.99 %) | 774934 (34.63 %) | 520323 (35.24 %) | 3465848 (34.06 %) |
|  |  |  |  |  |  |
| 40 weeks | 705100  (24.44 %) | 793865 (22.20 %) | 506116 (22.62 %) | 296847 (20.10 %) | 2301928 (22.62 %) |
|  |  |  |  |  |  |
| 41 weeks | 315990  (10.95 %) | 343941 (9.62 %) | 217561  (9.72 %) | 121217 (8.21 %) | 998709  (9.81 %) |
|  |  |  |  |  |  |
| 42 weeks | 89527  (3.10 %) | 116218  (3.25 %) | 69286  (3.10 %) | 46551  (3.15 %) | 321582  (3.16 %) |
|  |  |  |  |  |  |
| Infants gender = male | 1469493 (50.93 %) | 1820740 (50.92 %) | 1139987 (50.94 %) | 752855 (50.99 %) | 5183075 (50.94 %) |
|  |  |  |  |  |  |
| Birth weight (N %) |  |  |  |  |  |
| < 1500 grams | 1270  (0.04 %) | 1942  (0.05 %) | 1300  (0.06 %) | 1067  (0.07 %) | 5579  (0.05 %) |
|  |  |  |  |  |  |
| 1500-1999 grams | 6908  (0.24 %) | 9469  (0.26 %) | 6317  (0.28 %) | 4967  (0.34 %) | 27661  (0.27 %) |
|  |  |  |  |  |  |
| 2000-2499 grams | 74135  (2.57 %) | 98088  (2.74 %) | 65636  (2.93 %) | 48547  (3.29 %) | 286406  (2.81 %) |
|  |  |  |  |  |  |
| 2500-2999 grams | 489769  (16.98 %) | 617289 (17.26 %) | 388163 (17.35 %) | 279371 (18.92 %) | 1774592 (17.44 %) |
|  |  |  |  |  |  |
| 3000-3499 grams | 1207793 (41.86 %) | 1504589 (42.08 %) | 927314 (41.44 %) | 617248 (41.80 %) | 4256944 (41.84 %) |
|  |  |  |  |  |  |
| 3500-3999 grams | 851038  (29.50 %) | 1042500 (29.15 %) | 654229 (29.24 %) | 409746 (27.75 %) | 2957513 (29.07 %) |
|  |  |  |  |  |  |
| 4000-4499 grams | 220155  (7.63 %) | 260800 (7.29 %) | 168325  (7.52 %) | 100354 (6.80 %) | 749634  (7.37 %) |
|  |  |  |  |  |  |
| > 4499 grams | 34123  (1.18 %) | 41187  (1.15 %) | 26538  (1.19 %) | 15304  (1.04 %) | 117152  (1.15 %) |
|  |  |  |  |  |  |
| Plurality (N %) |  |  |  |  |  |
| Single | 2832295 (98.17 %) | 3520225 (98.44 %) | 2201579 (98.38 %) | 1454701 (98.52 %) | 1.00e+07 (98.36 %) |
|  |  |  |  |  |  |
| Twin | 52615  (1.82 %) | 55283  (1.55 %) | 36018  (1.61 %) | 21792  (1.48 %) | 165708  (1.63 %) |
|  |  |  |  |  |  |
| Triplet | 276  (0.01 %) | 333  (0.01 %) | 199  (0.01 %) | 103  (0.01 %) | 911  (0.01 %) |
|  |  |  |  |  |  |
| Quadruplet | 5 (0.00 %) | 23 (0.00 %) | 17 (0.00 %) | 7 (0.00 %) | 52 (0.00 %) |
|  |  |  |  |  |  |
| Quintuplet or higher | 0 (0.00 %) | 0 (0.00 %) | 9 (0.00 %) | 1 (0.00 %) | 10 (0.00 %) |
|  |  |  |  |  |  |
| Mother's Race (N %) |  |  |  |  |  |
| White | 2144049 (74.31 %) | 2884762 (80.67 %) | 1694000 (75.70 %) | 1127452 (76.35 %) | 7850263 (77.15 %) |
|  |  |  |  |  |  |
| Black | 358043  (12.41 %) | 458310 (12.82 %) | 428585 (19.15 %) | 289728 (19.62 %) | 1534666 (15.08 %) |
|  |  |  |  |  |  |
| American Indian/Alaskan Native | 15005  (0.52 %) | 42960  (1.20 %) | 31384  (1.40 %) | 25475  (1.73 %) | 114824  (1.13 %) |
|  |  |  |  |  |  |
| Asian/Pacific Islander | 368094  (12.76 %) | 189832 (5.31 %) | 83853  (3.75 %) | 33949  (2.30 %) | 675728  (6.64 %) |

**S1 Table: Sample Characteristics**
