# Supplementary material for: Causes of death and infant mortality rates among full-term births in the United States between 2010 and 2012: An observational study
Source: PLoS Med. 2018 Mar 20;15(3):e1002531. doi: 10.1371/journal.pmed.1002531 (PMC5860700; doi:10.1371/journal.pmed.1002531)
Supplement: S2 Table — (DOCX) [file pmed.1002531.s010.docx]

| U.S. States | Overall full-term infant mortality | Full-term infant mortality due to congenital malformations | Full-term infant mortality due to perinatal conditions | Full-term infant mortality due to SUDI | Full-term infant mortality due to other causes | Total number of live births |
| --- | --- | --- | --- | --- | --- | --- |
| CT | 12.88 | 3.17 | 1.78 | 5.45 | 2.48 | 100,900 |
| NJ | 13.15 | 3.71 | 1.82 | 5.15 | 2.46 | 263,943 |
| NH | 13.69 | 2.62 | 3.2 | 5.83 | 2.04 | 34,331 |
| MA | 14.78 | 4.68 | 1.63 | 6.05 | 2.42 | 190,124 |
| HI | 15.73 | 2.76 | 4.25 | 5.74 | 2.98 | 47,058 |
| NY | 15.86 | 5.64 | 2.14 | 5.48 | 2.6 | 629,695 |
| VT | 16.07 | 2.01 | 1.34 | 8.7 | 4.02 | 14,938 |
| CA | 16.68 | 6.64 | 2.02 | 5.37 | 2.65 | 1,337,504 |
| NV | 16.7 | 5.76 | 1.73 | 6.56 | 2.65 | 86,844 |
| MD | 17.29 | 4.06 | 2.95 | 7.28 | 3 | 179,854 |
| RI | 17.98 | 4.33 | 1 | 9.66 | 3 | 30,027 |
| MN | 19.69 | 6.86 | 2.25 | 6.98 | 3.6 | 177,764 |
| CO | 20.47 | 7.1 | 2.68 | 8.03 | 2.68 | 171,924 |
| IA | 20.55 | 5.87 | 2.02 | 9.31 | 3.34 | 98,785 |
| IL | 20.72 | 5.55 | 2.17 | 9.31 | 3.69 | 401,578 |
| WA | 20.99 | 6.54 | 2.52 | 9.19 | 2.74 | 226,319 |
| NM | 21.19 | 7.36 | 2.65 | 7.8 | 3.38 | 67,963 |
| OR | 21.32 | 6.1 | 3.51 | 9.78 | 1.92 | 119,590 |
| AK | 21.43 | 4.08 | 1.7 | 10.88 | 4.76 | 29,402 |
| FL | 21.51 | 6.08 | 2.25 | 10 | 3.17 | 532,857 |
| ID | 21.54 | 6.32 | 3.08 | 8.89 | 3.25 | 58,508 |
| UT | 21.57 | 8.09 | 3.06 | 7.8 | 2.62 | 137,236 |
| WI | 21.58 | 5.72 | 2.23 | 9.27 | 4.35 | 174,685 |
| DC | 21.79 | 9.46 | 3.15 | 6.02 | 3.15 | 34,877 |
| NE | 21.79 | 9.34 | 1.93 | 7.86 | 2.67 | 67,447 |
| TX | 21.91 | 7.47 | 2.56 | 8.96 | 2.92 | 990,101 |
| VA | 22.35 | 7.36 | 2.45 | 9.15 | 3.39 | 256,801 |
| AZ | 22.54 | 8.34 | 2.39 | 8.3 | 3.52 | 221,820 |
| GA | 23.07 | 5.95 | 2.61 | 11.4 | 3.11 | 341,208 |
| MI | 23.36 | 6.61 | 2.9 | 10.77 | 3.08 | 286,002 |
| KS | 23.55 | 6 | 2.06 | 12.01 | 3.47 | 106,582 |
| PA | 24.29 | 7.77 | 2.96 | 9.89 | 3.67 | 368,108 |
| NC | 25.16 | 8.2 | 2.33 | 10.73 | 3.9 | 304,849 |
| ND | 25.38 | 4.23 | 2.47 | 16.21 | 2.47 | 28,373 |
| MT | 25.99 | 4.55 | 2.92 | 16.25 | 2.27 | 30,778 |
| SC | 26.69 | 6.44 | 2.85 | 13.75 | 3.66 | 136,742 |
| MO | 27.29 | 9.08 | 3.08 | 11.69 | 3.44 | 194,956 |
| IN | 27.38 | 8.29 | 3.48 | 11.08 | 4.53 | 218,404 |
| WY | 27.55 | 3.86 | 3.86 | 15.43 | 4.41 | 18,151 |
| ME | 27.62 | 7.06 | 2.45 | 14.73 | 3.38 | 32,590 |
| DE | 28.31 | 7.25 | 4.14 | 9.67 | 7.25 | 28,960 |
| KY | 28.53 | 8.11 | 1.95 | 14.19 | 4.28 | 133,180 |
| OH | 29.04 | 9.03 | 2.89 | 13.1 | 4.02 | 353,314 |
| SD | 29.27 | 7.78 | 4.36 | 12.45 | 4.67 | 32,117 |
| WV | 31.1 | 7.92 | 4.34 | 15.64 | 3.2 | 53,054 |
| TN | 31.23 | 8.33 | 2.56 | 17.17 | 3.16 | 214,860 |
| AL | 31.23 | 8.48 | 3.32 | 15.41 | 4.03 | 141,508 |
| OK | 31.27 | 8.54 | 2.33 | 17.15 | 3.26 | 128,873 |
| LA | 31.52 | 7.52 | 2.22 | 15.43 | 6.34 | 152,924 |
| AR | 33.54 | 9.63 | 2.43 | 17.46 | 4.02 | 94,528 |
| MS | 37.71 | 9.4 | 4 | 19.88 | 4.43 | 92,54 |

*Notes*: Based on the pooled sample covering all full-term infants born in the United States between 2010 and 2012. The following ICD-10 causes of death were included in each group: Congenital malformations: Q00-Q99; SUDI: V01-Y89 and R00-R99, Perinatal conditions: P00-P96; Other: all other causes.
