# Supplementary material for: Causes of death and infant mortality rates among full-term births in the United States between 2010 and 2012: An observational study
Source: PLoS Med. 2018 Mar 20;15(3):e1002531. doi: 10.1371/journal.pmed.1002531 (PMC5860700; doi:10.1371/journal.pmed.1002531)
Supplement: S3 Table — (DOCX) [file pmed.1002531.s011.docx]

**S3 Table: Cause-specific Mortality Distribution by Mortality Groups**

| FTIMR Group |  | All cause full term infant mortality | Full term infant mortality due to congenital malformations | Full term infant mortality due to perinatal conditions | Full term infant mortality due to SUDI | Full term infant mortality due to other causes |
| --- | --- | --- | --- | --- | --- | --- |
|  |  |  |  |  |  |  |
| Good | Median | 1.67 | 0.56 | 0.20 | 0.54 | 0.27 |
|  | IQR | 0.08 | 0.20 | 0.01 | 0.01 | 0.00 |
|  | Minimum | 1.29 | 0.20 | 0.13 | 0.52 | 0.20 |
|  | Maximum | 1.73 | 0.66 | 0.43 | 0.87 | 0.40 |
|  |  |  |  |  |  |  |
| Average | Median | 2.15 | 0.69 | 0.25 | 0.92 | 0.32 |
|  | IQR | 0.09 | 0.14 | 0.03 | 0.04 | 0.05 |
|  | Minimum | 1.80 | 0.41 | 0.10 | 0.60 | 0.19 |
|  | Maximum | 2.24 | 0.95 | 0.35 | 1.09 | 0.48 |
|  |  |  |  |  |  |  |
| Fair | Median | 2.43 | 0.78 | 0.29 | 1.08 | 0.35 |
|  | IQR | 0.29 | 0.19 | 0.06 | 0.15 | 0.06 |
|  | Minimum | 2.25 | 0.42 | 0.21 | 0.83 | 0.23 |
|  | Maximum | 2.74 | 0.91 | 0.35 | 1.62 | 0.45 |
|  |  |  |  |  |  |  |
| Poor | Median | 3.12 | 0.85 | 0.26 | 1.54 | 0.40 |
|  | IQR | 0.22 | 0.09 | 0.06 | 0.41 | 0.10 |
|  | Minimum | 2.75 | 0.39 | 0.20 | 0.97 | 0.32 |
|  | Maximum | 3.77 | 0.96 | 0.44 | 1.99 | 0.73 |
|  |  |  |  |  |  |  |
| Total | Median | 2.16 | 0.66 | 0.24 | 0.92 | 0.31 |
|  | IQR | 0.76 | 0.18 | 0.07 | 0.47 | 0.10 |
|  | Minimum | 1.29 | 0.20 | 0.10 | 0.52 | 0.19 |
|  | Maximum | 3.77 | 0.96 | 0.44 | 1.99 | 0.73 |
| Table shows state-level cause-specific mortality rates by FTIMR group. All estimates correspond to infant deaths per 1000 full-term live births. Numbers reported are average state-level estimates based on the full sample period 2010-2012. | | | | | | |
