# Supplementary material for: Causes of death and infant mortality rates among full-term births in the United States between 2010 and 2012: An observational study
Source: PLoS Med. 2018 Mar 20;15(3):e1002531. doi: 10.1371/journal.pmed.1002531 (PMC5860700; doi:10.1371/journal.pmed.1002531)
Supplement: S4 Table — (DOCX) [file pmed.1002531.s012.docx]

**S4 Table: Estimates from Random Intercept Logistic Model (Odds Ratio)**

| Outcome | All cause full term infant mortality | Full term infant mortality due to congenital malformations | Full term infant mortality due to perinatal conditions | Full term infant mortality due to SUDI | Full term infant mortality due to other causes |
| --- | --- | --- | --- | --- | --- |
| < High School | Reference | | | | |
|  |  |  |  |  |  |
| High School/College Credit | 0.814*** | 0.753*** | 0.947 | 0.863*** | 0.725*** |
|  | (0.784,0.845) | (0.703,0.807) | (0.837,1.071) | (0.817,0.913) | (0.656,0.801) |
| Associate/Bachelor's Degree | 0.565*** | 0.661*** | 0.899 | 0.384*** | 0.598*** |
|  | (0.537,0.595) | (0.607,0.719) | (0.776,1.042) | (0.350,0.420) | (0.527,0.680) |
| Master's Degree/Doctorate | 0.444*** | 0.481*** | 0.725** | 0.259*** | 0.581*** |
|  | (0.411,0.481) | (0.424,0.546) | (0.591,0.890) | (0.220,0.305) | (0.487,0.693) |
| Diabetes = Yes | 0.918* | 0.838** | 0.858 | 1.085 | 0.832* |
|  | (0.859,0.981) | (0.751,0.935) | (0.712,1.034) | (0.968,1.216) | (0.705,0.981) |
| Chronic Hypertension = Yes | 0.847** | 1.026 | 0.782 | 0.713*** | 0.890 |
|  | (0.756,0.948) | (0.841,1.251) | (0.565,1.084) | (0.600,0.849) | (0.652,1.216) |
| Eclampsia = Yes | 1.407 | 2.180* | 2.803 | 0.860 | 1.128 |
|  | (0.996,1.988) | (1.167,4.073) | (0.698,11.260) | (0.526,1.407) | (0.468,2.718) |
| Cigarettes in 1st Trimester = Yes | 1.237*** | 0.818 | 0.922 | 1.730*** | 1.108 |
|  | (1.108,1.381) | (0.650,1.028) | (0.632,1.346) | (1.490,2.007) | (0.817,1.502) |
| Cigarettes in 2nd Trimester = Yes | 1.374*** | 0.909 | 1.341 | 1.553*** | 1.793** |
|  | (1.163,1.622) | (0.629,1.312) | (0.755,2.379) | (1.245,1.936) | (1.150,2.795) |
| Cigarettes in 3rd Trimester = Yes | 1.063 | 0.813 | 0.939 | 1.326** | 0.712 |
|  | (0.924,1.223) | (0.592,1.118) | (0.578,1.524) | (1.103,1.594) | (0.494,1.026) |
| Mother's Age < 20 | 1.120*** | 0.807*** | 1.281** | 1.303*** | 1.109 |
|  | (1.067,1.176) | (0.731,0.891) | (1.100,1.492) | (1.218,1.395) | (0.974,1.263) |
| Age 20-34 | Reference | | | | |
|  |  |  |  |  |  |
| Age 35-39 | 0.944* | 1.287*** | 1.048 | 0.594*** | 0.960 |
|  | (0.893,0.997) | (1.184,1.400) | (0.906,1.213) | (0.531,0.663) | (0.838,1.100) |
| Age 40-44 | 1.227*** | 2.153*** | 1.204 | 0.528*** | 0.751 |
|  | (1.118,1.347) | (1.906,2.432) | (0.929,1.560) | (0.417,0.667) | (0.559,1.007) |
| Age > 44 | 1.782*** | 3.374*** | 0.692 | 0.356 | 1.664 |
|  | (1.338,2.374) | (2.402,4.739) | (0.222,2.157) | (0.115,1.106) | (0.790,3.503) |
| Mother’s race = Black | 1.323*** | 0.853*** | 1.089 | 1.868*** | 1.308*** |
|  | (1.271,1.377) | (0.790,0.921) | (0.962,1.234) | (1.764,1.979) | (1.178,1.452) |
| American Indian / Alaskan Native | 1.619*** | 1.146 | 1.151 | 2.028*** | 1.487* |
|  | (1.442,1.817) | (0.895,1.468) | (0.766,1.729) | (1.741,2.362) | (1.081,2.045) |
| Asian / Pacific Islander | 0.791*** | 0.633*** | 0.739** | 0.845* | 0.990 |
|  | (0.732,0.856) | (0.558,0.719) | (0.594,0.919) | (0.731,0.976) | (0.830,1.182) |
| Gestation Age 37 weeks | 1.188*** | 1.069 | 1.429*** | 1.208*** | 1.261*** |
|  | (1.135,1.244) | (0.990,1.154) | (1.250,1.634) | (1.123,1.300) | (1.118,1.421) |
| 38 weeks | 1.116*** | 1.115** | 1.153* | 1.105** | 1.115* |
|  | (1.074,1.161) | (1.040,1.194) | (1.023,1.299) | (1.040,1.174) | (1.007,1.234) |
| 39-40 weeks | Reference | | | | |
|  |  |  |  |  |  |
| 41 weeks | 1.110*** | 1.186** | 1.213* | 1.044 | 1.079 |
|  | (1.049,1.174) | (1.069,1.316) | (1.034,1.423) | (0.959,1.137) | (0.934,1.246) |
| 42 weeks | 1.124** | 1.111 | 1.306* | 1.137 | 0.932 |
|  | (1.030,1.228) | (0.942,1.309) | (1.024,1.667) | (0.999,1.294) | (0.727,1.194) |
| Gender = Male | 1.324*** | 1.225*** | 1.126* | 1.412*** | 1.421*** |
|  | (1.284,1.366) | (1.160,1.294) | (1.027,1.234) | (1.346,1.481) | (1.310,1.540) |
| Birth Weight < 1500 grams | 38.455*** | 126.172*** | 41.691*** | 4.097*** | 20.049*** |
|  | (31.646,46.730) | (87.697,181.526) | (29.051,59.830) | (2.399,6.995) | (11.747,34.219) |
| 1500-1999 grams | 18.745*** | 103.276*** | 4.003*** | 2.734*** | 8.121*** |
|  | (15.814,22.218) | (73.873,144.383) | (2.661,6.020) | (1.904,3.926) | (5.128,12.862) |
| 2000-2499 grams | 4.316*** | 16.559*** | 1.375 | 1.996*** | 3.006*** |
|  | (3.678,5.065) | (11.902,23.040) | (0.983,1.921) | (1.510,2.637) | (2.016,4.482) |
| 2500-2999 grams | 1.546*** | 3.139*** | 0.565*** | 1.417* | 1.387 |
|  | (1.324,1.807) | (2.262,4.358) | (0.414,0.771) | (1.086,1.848) | (0.947,2.033) |
| 3000-3499 grams | 0.911 | 1.134 | 0.367*** | 1.107 | 0.899 |
|  | (0.781,1.063) | (0.817,1.574) | (0.271,0.496) | (0.851,1.440) | (0.616,1.312) |
| 3500-3999 grams | 0.677*** | 0.643** | 0.340*** | 0.898 | 0.715 |
|  | (0.579,0.792) | (0.461,0.897) | (0.250,0.463) | (0.689,1.171) | (0.488,1.048) |
| 4000-4499 grams | 0.658*** | 0.598** | 0.395*** | 0.856 | 0.727 |
|  | (0.556,0.779) | (0.415,0.860) | (0.280,0.557) | (0.646,1.136) | (0.482,1.095) |
| > 4499 grams | Reference | | | | |
|  |  |  |  |  |  |
| Birth Outcome = Single | Reference | | | | |
|  |  |  |  |  |  |
| Twin | 0.678*** | 0.322*** | 1.385** | 1.144 | 0.751* |
|  | (0.616,0.745) | (0.273,0.380) | (1.120,1.713) | (0.971,1.348) | (0.577,0.976) |
| Triplet | 1.109 | 0.167* | 3.120** | 2.751 | 1.801 |
|  | (0.633,1.940) | (0.042,0.674) | (1.427,6.819) | (0.680,11.133) | (0.443,7.331) |
| Quadruplet | 0.919 | Dropped | | | 10.375* |
|  | (0.122,6.911) |  |  |  | (1.343,80.144) |
| Quintuplet or higher | Dropped | | | | |
|  |  |  |  |  |  |
| Observations (N) | 7,749,466 | 7,749,430 | 7,749,430 | 7,749,430 | 7,749,466 |

Notes: Table shows results from multivariable multi-level logistic models where individuals are nested within states, and state level variation is modeled through state specific random intercepts. Displayed coefficients are odds rations with 95% confidence intervals in parentheses. * p < 0.10, ** p < 0.05, *** p< 0.01.
