# Supplementary material for: Causes of death and infant mortality rates among full-term births in the United States between 2010 and 2012: An observational study
Source: PLoS Med. 2018 Mar 20;15(3):e1002531. doi: 10.1371/journal.pmed.1002531 (PMC5860700; doi:10.1371/journal.pmed.1002531)
Supplement: S5 Table — (DOCX) [file pmed.1002531.s013.docx]

**S5 Table: Odds Ratio of cause-specific full-term infant mortality for children born in states with poor vs. children born in states with good FTIMR**

| Outcome | Full term infant mortality due to congenital malformations | Full term infant mortality due to perinatal conditions | Full term infant mortality due to SUDI | Full term infant mortality due to other causes |
| --- | --- | --- | --- | --- |
| Born in a state with FTIMR < 1.75 | Reference | | | |
| Born in a state with FTIMR > 3.75 | 1.37*** | 1.16 | 1.70*** | 1.37*** |
|  | (1.19,1.58) | (0.97,1.38) | (1.48,1.94) | (1.15,1.64) |
| < High School | Reference | | | |
| High School/College Credit | 0.74*** | 0.91 | 0.89 | 0.69*** |
|  | (0.66,0.82) | (0.74,1.10) | (0.79,1.01) | (0.60,0.79) |
| Associate/Bachelor's Degree | 0.59*** | 0.84 | 0.41*** | 0.54*** |
|  | (0.51,0.68) | (0.63,1.13) | (0.35,0.48) | (0.45,0.64) |
| Master's Degree/Doctorate | 0.44*** | 0.68* | 0.29*** | 0.51*** |
|  | (0.38,0.51) | (0.47,0.97) | (0.25,0.33) | (0.44,0.60) |
| Diabetes = Yes | 0.75*** | 0.88 | 1.12 | 0.93 |
|  | (0.64,0.88) | (0.69,1.13) | (0.96,1.31) | (0.70,1.23) |
| Chronic Hypertension = Yes | 0.81 | 0.71 | 0.86 | 0.68 |
|  | (0.57,1.16) | (0.41,1.25) | (0.68,1.10) | (0.46,1.00) |
| Eclampsia = Yes | 1.47 | Dropped | 0.61* | 2.79 |
|  | (0.82,2.66) |  | (0.40,0.93) | (0.35,22.23) |
| Cigarettes in 1st Trimester = Yes | 0.70 | 0.73 | 1.84* | 0.98 |
|  | (0.45,1.09) | (0.28,1.92) | (1.15,2.94) | (0.46,2.06) |
| Cigarettes in 2nd Trimester = Yes | 0.99 | 1.55 | 1.51* | 1.55 |
|  | (0.56,1.75) | (0.31,7.81) | (1.07,2.13) | (0.41,5.87) |
| Cigarettes in 3rd Trimester = Yes | 0.84 | 0.96 | 1.29 | 0.95 |
|  | (0.55,1.27) | (0.25,3.66) | (0.75,2.23) | (0.44,2.05) |
| Mother's Age < 20 | 0.74*** | 1.53*** | 1.41*** | 1.04 |
|  | (0.62,0.88) | (1.28,1.83) | (1.21,1.65) | (0.82,1.31) |
| Age 20-34 | Reference | | | |
| Age 35-39 | 1.18*** | 0.98 | 0.60*** | 0.90 |
|  | (1.09,1.27) | (0.81,1.19) | (0.55,0.65) | (0.79,1.02) |
| Age 40-44 | 1.83*** | 1.45 | 0.44*** | 0.65 |
|  | (1.43,2.34) | (0.98,2.14) | (0.31,0.62) | (0.35,1.19) |
| Age > 44 | 2.89*** | 0.45 | 1.00 | 1.84 |
|  | (2.16,3.86) | (0.11,1.93) | (1.00,1.00) | (0.84,4.04) |
| Mother’s race = Black | 0.73*** | 1.08 | 1.73*** | 1.24** |
|  | (0.61,0.87) | (0.91,1.27) | (1.40,2.14) | (1.05,1.46) |
| American Indian / Alaskan Native | 0.97 | 1.02 | 2.33*** | 1.63** |
|  | (0.68,1.38) | (0.47,2.21) | (1.71,3.18) | (1.18,2.25) |
| Asian / Pacific Islander | 0.68*** | 0.86 | 0.89 | 1.00 |
|  | (0.60,0.76) | (0.70,1.07) | (0.65,1.21) | (0.84,1.20) |
| Gestation Age 37 weeks | 1.10 | 1.55*** | 1.20*** | 1.29* |
|  | (0.97,1.25) | (1.25,1.91) | (1.08,1.33) | (1.03,1.63) |
| 38 weeks | 1.16** | 1.30** | 1.09* | 1.03 |
|  | (1.04,1.29) | (1.10,1.54) | (1.00,1.18) | (0.92,1.16) |
| 39-40 weeks | Reference | | | |
| 41 weeks | 1.17 | 1.16 | 0.98 | 1.13 |
|  | (0.96,1.43) | (0.95,1.40) | (0.90,1.08) | (0.95,1.35) |
| 42 weeks | 1.14 | 1.67* | 1.09 | 1.08 |
|  | (0.88,1.49) | (1.01,2.75) | (0.92,1.28) | (0.73,1.58) |
| Gender = Male | 1.23*** | 1.20* | 1.42*** | 1.36*** |
|  | (1.14,1.32) | (1.04,1.38) | (1.33,1.51) | (1.18,1.57) |
| Birth Weight < 1500 grams | 132.97*** | 42.93*** | 4.49*** | 32.87*** |
|  | (78.34,225.69) | (17.93,102.82) | (2.03,9.95) | (14.29,75.63) |
| 1500-1999 grams | 103.22*** | 2.71*** | 2.56** | 13.66*** |
|  | (65.66,162.27) | (1.81,4.05) | (1.38,4.73) | (6.44,28.96) |
| 2000-2499 grams | 17.32*** | 1.78* | 2.38** | 5.15*** |
|  | (11.21,26.76) | (1.09,2.93) | (1.34,4.24) | (2.78,9.54) |
| 2500-2999 grams | 3.20*** | 0.60* | 1.59 | 2.24** |
|  | (1.64,6.24) | (0.40,0.91) | (0.98,2.58) | (1.34,3.75) |
| 3000-3499 grams | 1.18 | 0.43*** | 1.31 | 1.48 |
|  | (0.61,2.30) | (0.26,0.69) | (0.80,2.16) | (0.87,2.52) |
| 3500-3999 grams | 0.79 | 0.42*** | 1.00 | 1.31 |
|  | (0.39,1.61) | (0.25,0.70) | (0.60,1.67) | (0.76,2.26) |
| 4000-4499 grams | 0.68 | 0.52** | 0.89 | 1.29 |
|  | (0.33,1.41) | (0.33,0.81) | (0.50,1.58) | (0.82,2.02) |
| > 4499 grams | Reference | | | |
| Birth Outcome = Single | Reference | | | |
| Twin | 0.30*** | 1.31 | 1.11 | 0.77 |
|  | (0.22,0.41) | (0.70,2.46) | (0.85,1.44) | (0.57,1.06) |
| Triplet | 0.25 | Dropped | 8.31 | 2.52 |
|  | (0.03,2.19) |  | (0.95,72.38) | (0.71,8.96) |
| Quadruplet | Dropped | | | |
|  |  |  |  |  |
| Number of observa-tions | 3,385,884 | 3,380,110 | 3,378,831 | 3,385,884 |

Notes: Table shows results from multivariable logistic models comparing children born in states with poor FTIMR (FTIMR > 2.75) to states with good FTIMR (< 1.75). Displayed coefficients are odds ratios with 95% confidence intervals in parentheses. Standard errors are clustered at the state level. * p < 0.10, ** p < 0.05, *** p< 0.01.
